# Multiple signaling kinases target Mrc1 to prevent genomic instability triggered by transcription-replication conflicts

**DOI:** 10.1038/s41467-017-02756-x

**Published:** 2018-01-25

**Authors:** Alba Duch, Berta Canal, Sonia I. Barroso, María García-Rubio, Gerhard Seisenbacher, Andrés Aguilera, Eulàlia de Nadal, Francesc Posas

**Affiliations:** 1 Cell Signaling Research Group, Departament de Ciències Experimentals i de la Salut, Universitat Pompeu Fabra (UPF), E-08003 Barcelona, Spain; 2 Centro Andaluz de Biología Molecular and Medicina Regenerativa-CABIMER, Universidad de Sevilla, 41092 Seville, Spain

## Abstract

Conflicts between replication and transcription machineries represent a major source of genomic instability and cells have evolved strategies to prevent such conflicts. However, little is known regarding how cells cope with sudden increases of transcription while replicating. Here, we report the existence of a general mechanism for the protection of genomic integrity upon transcriptional outbursts in S phase that is mediated by Mrc1. The N-terminal phosphorylation of Mrc1 blocked replication and prevented transcription-associated recombination (TAR) and genomic instability during stress-induced gene expression in S phase. An unbiased kinome screening identified several kinases that phosphorylate Mrc1 at the N terminus upon different environmental stresses. Mrc1 function was not restricted to environmental cues but was also required when unscheduled transcription was triggered by low fitness states such as genomic instability or slow growth. Our data indicate that Mrc1 integrates multiple signals, thereby defining a general safeguard mechanism to protect genomic integrity upon transcriptional outbursts.

## Abstract (web summary)

During S phase of the cell cycle, transcription and replication need to be coordinated in order to avoid conflicts leading to potential genomic instability. Here, the authors find that Mrc1 integrates signals from different kinases to regulate replication during unscheduled transcription events.

## Introduction

Transcription–replication conflicts are a major source of genomic instability^1,2^. During S phase, transcription coexists in time and space with DNA replication, and therefore, the two processes must be coordinated to prevent transcription–replication conflicts. S phase is the period of the cell cycle that is the most susceptible to the accumulation of DNA lesions because the unwrapped structure of chromatin in S phase makes DNA more vulnerable to internal and external mutagenic agents^3^. Moreover, the DNA replication machinery must cope with multiple obstacles that impede replication fork progression leading to double-strand breaks (DSBs) and unscheduled recombination events that challenge genomic integrity^4,5^. One of the most important blocks that the replisome must overcome is the transcription machinery. The collision between replication and transcription machineries results in replication fork stalling that leads to transcription-associated recombination (TAR) and genomic instability. These phenomena highlight the relevance of coordinating replication and transcription for maintaining genomic integrity^1,2,6–12^.

Cells are constantly exposed to environmental changes. The maintenance of cell viability upon sudden changes in osmolarity, temperature, pH, nutrient supply, or oxidative stress is critical for any living organism. To cope with these changes, cells have evolved sophisticated signal transduction pathways that control many aspects of cell physiology, including the control of gene expression^13,14^. For instance, yeast cells trigger a common transcriptional response called the environmental stress response (ESR) when exposed to a wide variety of environmental stresses^15^. This transcriptional program consists of the rapid induction of more than 300 genes that play roles in many physiological functions. Although the ESR is essential for maximizing cell fitness, such massive changes in gene expression pose a risk to genomic integrity when they coincide with DNA replication.

In response to osmostress, the yeast Hog1 MAPK induces hundreds of osmoresponsive genes^16,17^ and, the induction of these osmoresponsive genes can also occur during S phase. In addition, Hog1 also directly prevents collisions between transcription and replication machineries by phosphorylating the N-terminal region of Mrc1 to block DNA replication. Mrc1 is a basic regulatory component of the replication complex that links the helicase with DNA polymerase activities^18–21^ and, it is crucial to maintain an adequate replication fork progression rate^18^. This phosphorylation prevents TAR and subsequent genomic instability upon osmostress^22,23^. Remarkably, this mechanism operates independently of the known DNA damage checkpoint pathway that responds to DNA damage and replication stress^24^, which points to the necessity of a dedicated S-phase control mechanism to deal with the massive transcription that occurs upon osmostress. Therefore, since other environmental stresses also induce massive changes in gene expression, which are not controlled by Hog1, there may be another mechanism(s) that protects genomic integrity and prevents transcription–replication conflicts upon these other stress-dependent transcriptional outbursts.

Here, we show that several stresses provoked a delay in S phase that was mediated by the N-terminal phosphorylation of Mrc1. Mrc1 was phosphorylated by several signaling kinases and its phosphorylation served to prevent TAR and genomic instability and to maximize cell viability. Of note, Mrc1 function was not restricted to environmental stresses but was also necessary to prevent TAR and genomic instability upon transcription triggered by mutations that compromise cell fitness. Therefore, we propose that there exists a general S-phase control mechanism that is mediated by Mrc1, which we call the “Mrc1 transcription–replication safeguard mechanism” (MTR), that serves to prevent genomic instability triggered by transcription–replication conflicts that are caused by unscheduled transcription during S phase.

## Results

### Mrc1 phosphorylation upon stress delays replication

**Fig. 1:**
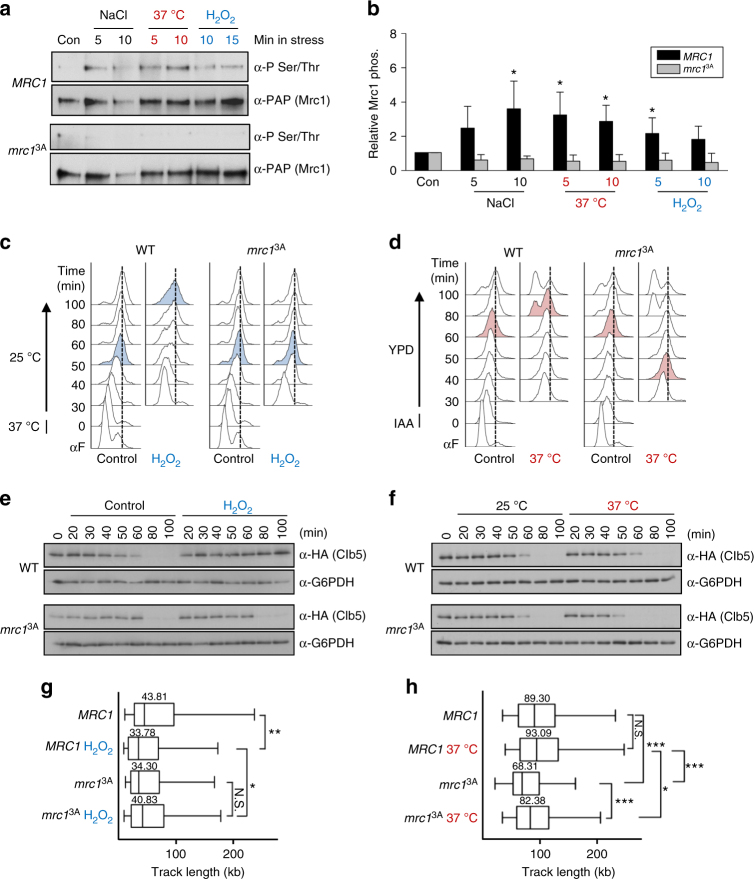
Mrc1 is phosphorylated upon stress to delay DNA replication. **a** Mrc1 is phosphorylated upon osmotic, heat, and oxidative stresses and *mrc1*^3A^ protein suppresses this phosphorylation. Mrc1–TAP and *mrc1*^3A^–TAP protein were immunoprecipitated (IP) from cells subjected to several stresses and phosphorylation was assessed by western blotting using α-phospho Ser/Thr antibodies. Total Mrc1–TAP protein levels were assessed using the anti-PAP antibody. Con control. **b** The graphs indicate the relative phosphorylation of Mrc1 and *mrc1*^3A^ (assessed as in **a**) normalized to the total amount of precipitated Mrc1–TAP protein and then referenced to the control conditions. The data represent the mean and standard deviation of three independent experiments. Values that are significantly different (respect to the control conditions) from Student's *t* test are indicated by asterisks (*p < 0.05). **c**, **d** Oxidative (**c**) and heat (**d**) stresses cause a delay in cell cycle progression that is abolished in the *mrc1*^3A^ cells. Cells were synchronized at the beginning of S phase as described (see “Methods” section) and released into S phase at 25 °C in YPD (control) or in the presence of oxidative (**c**) or heat stress (**d**). DNA replication was assessed using FACS. The vertical dotted lines indicate the end of S phase (2C peak) and the colored plots indicate the time point at which cells have reached the 2C peak. IAA indole acetic acid, α-F pheromone (**e**, **f**) *mrc1*^*3A*^ cells do not delay Clb5 degradation upon oxidative (**e**) or heat (**f**) stress, in contrast to wild-type cells. Cells were treated as in **c** and **d** and analyzed using western blotting. α-G6PDH, loading control. **g**, **h** Replication fork progression is slowed down upon stress. DNA from cells treated as in **c** and **d** was assessed by combing analysis of replication forks in WT and *mrc1*^*3A*^ cells in control conditions or upon oxidative and heat stresses, as indicated, after their release into S phase. The graphs indicate the distribution of BrdU track length (kb) upon oxidative and heat stress during 30 and 15 min of labeling, respectively. Box, 25–75 percentile range; Whiskers, 5–95 percentile range; *Values significantly different (*p < 0.05, **p < 0.01) as determined by the Mann–Whitney *U* test

Several stresses (e.g., heat or oxidative stress) elicit significant changes in gene expression (ESR) similar to those observed upon osmostress. In yeast, Mrc1 is phosphorylated by Hog1 upon osmostress^23^. To assess whether Mrc1 is phosphorylated in vivo in response to other stresses, Mrc1–TAP was immunoprecipitated from cells subjected to osmotic, heat, or oxidative stress and its phosphorylation was analyzed by western blotting with anti-pSer/Thr antibodies. Mrc1 was phosphorylated under all stress conditions tested (Fig. 1a). This phosphorylation occurred on the Hog1-specific phosphorylation sites (T169, S215, and S229) since it was abolished in cells carrying a mutant allele in these three sites (*mrc1*^*3A*^) (Fig. 1a, b). Thus, different stresses elicit the phosphorylation of the same N-terminal sites in Mrc1. Of note, this phosphorylation did not cause a mobility shift neither in wild-type nor in *mrc1*^*3A*^ cells, while it did in HU-treated cells, suggesting that HU promotes phosphorylation of alternative sites different from those caused by stress (Supplementary Figure 1A).

We next assessed the relevance of the N-terminal phosphorylation of Mrc1 upon different stresses for cell cycle progression. For analysis of H_2_O_2_ stress, the cells were synchronized at the onset of S phase by presynchronizing a temperature-sensitive *CDC7* allele (*cdc7*^*ts4*^) using pheromone and the cells were subsequently released from this S-phase block at permissive temperature into media with or without H_2_O_2_. S-phase progression was delayed upon oxidative stress compared to nonstressed cells and this delay was not observed in cells carrying the nonphosphorylatable *MRC1* allele, *mrc1*^*3A*^ (Fig. 1c). For analysis of heat stress, pheromone presynchronized cells were arrested at S-phase onset using the *cdc7*^*AID*^ (auxin-induced degron) system before their release at 25 °C or at 37 °C (heat stress). As expected, both the *MRC1* wild-type and mutant strains progressed faster into S phase at 37 °C than at 25 °C; however, cells carrying the *mrc1*^*3A*^ allele progressed faster than wild-type cells suggesting that phosphorylation of Mrc1 leads to a delay in replication upon heat stress (Fig. 1d). Clb5 is degraded after cells exit the S phase^25,26^. Of note, no differences in cell cycle progression were observed for the *mrc1*^*3A*^ cells when compared to wild type in the absence of stress in contrast to mrc1 cells that progressed more slowly (Supplementary Figure 2). To support the FACS data, we therefore monitored Clb5 protein expression under the same experimental conditions using cells expressing endogenously HA-tagged Clb5. Wild-type cells subjected to oxidative stress showed a clear delay in Clb5 degradation that was not observed in *mrc1*^*3A*^ cells (Fig. 1e). Similarly, Clb5 was degraded earlier in *mrc1*^*3A*^ cells subjected to heat stress than in wild-type cells (Fig. 1f). Of note, HU induces a slight increase on Mrc1 phosphorylation since multiple sites (AQ/TQ), different from those targeted by Hog1, are known to be phosphorylated by Mec1 in response to HU. However, HU stimulates a clear mobility shift in a PAGE gel on Mrc1 that occurs both in wild-type and *mrc1*^*3A*^ cells^23^ which was not observed upon osmostress (Fig. 1a and Supplementary Figure 1A). Furthermore, we assessed cell cycle progression in *mrc1*^*AQ*^ (a mutant that contains the Mec1 sites mutated to alanine) and the *mrc1*^*3A*^ in response to replication stress (HU) and osmostress. We found that while *mrc1*^*3A*^ cells were arrested upon HU, they did not arrest upon osmostress and in contrast, *mrc1*^*AQ*^ cells were competent to arrest upon osmostress but not in response to HU (Supplementary Figure 1B and Supplementary Figure 1C). These results supported that N-terminal phosphorylation of Mrc1 delays S-phase progression upon oxidative and heat stresses as it does for osmostress.

To further determine that N-terminal phosphorylation of Mrc1 delays DNA replication upon heat or oxidative stress, we performed DNA-combing experiments using synchronous cultures to measure replication fork (RF) progression (track length). In contrast to *mrc1*^*3A*^ cells, wild-type cells showed significantly shorter replication tracks upon oxidative stress, indicating a delay in RF progression (Fig. 1g). Upon heat stress, wild-type cells showed similar RF progression at 25 °C and 37 °C in contrast to *mrc1*^*3A*^ cells in which the RF progressed faster at 37 °C (Fig. 1h). Of note, the track length in both wild-type and *mrc1*^*3A*^ cells was significantly higher than that in Fig. 1g, suggesting the possibility of fusions of adjacent replicons. These data indicated that the delay observed in cell cycle progression upon oxidative and heat stresses due to Mrc1 phosphorylation was caused by altered DNA replication.

### Mrc1 phosphorylation prevents transcription–replication conflicts

**Fig. 2:**
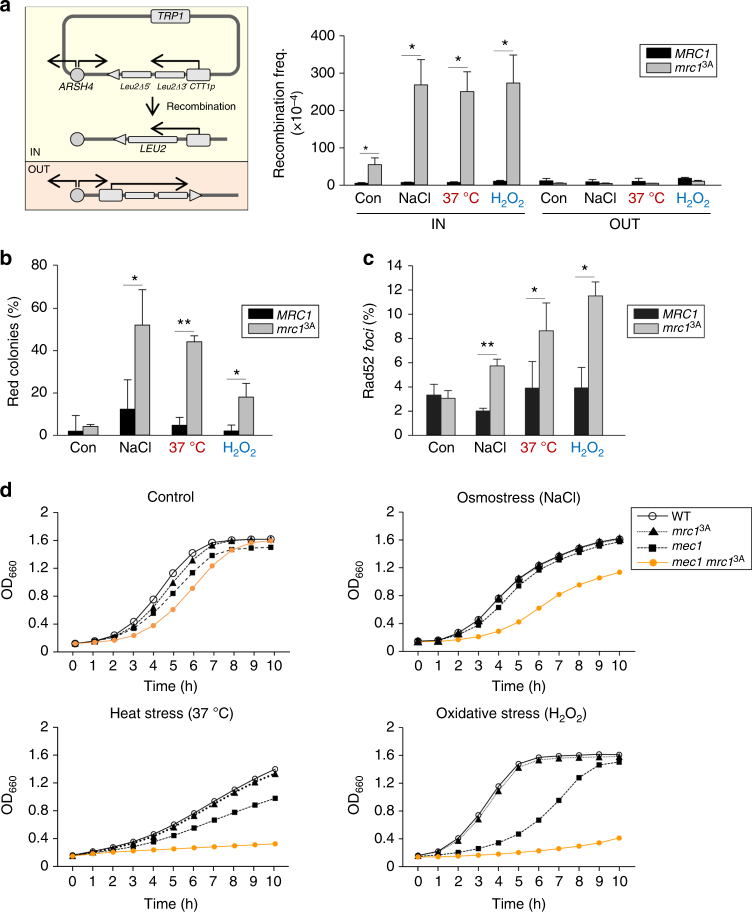
Mrc1 phosphorylation prevents TAR and genomic instability. **a** Schematic diagram of the IN/OUT vectors used in the TAR assays. *mrc1*^3A^ cells display higher levels of recombination than wild-type cells in TAR assays upon release of synchronized cells from S-phase block into osmotic, heat stress, and oxidative stresses (see Methods). **b** *mrc1*^3A^ cells show higher frequency of plasmid loss upon release of synchronized cells from S-phase block into environmental stresses in a red-sectoring assay (see “Methods” section). **c** *mrc1*^3A^ cells display a higher percentage of Rad52-YFP *foci* upon release of synchronized cells from S-phase block into environmental stresses. Data in **a**, **b**, and **c** represent the mean and standard deviation of three independent experiments. Values that are significantly different (*p < 0.05, **p < 0.01, ***p < 0.005) from Student's *t* test are annotated. **d** *mrc1*^3A^ cells show a synthetic cell growth defect with *mec1* deletion upon stress. The indicated strains were grown to log phase and subjected to the indicated stresses

Induction of gene expression is a major adaptive response to stress. When this transcription is coincident with ongoing replication, it could provoke transcription–replication conflicts resulting in TAR and genomic instability^2,27^. We therefore asked whether TAR increased upon oxidative or heat stress. To measure TAR, we assessed the recombination of a *leu2* direct repeat whose transcription was driven by the *CTT1* stress-responsive promoter, a prototypical gene of the ESR that responds to osmo, heat, and oxidative stress, which was oriented IN or OUT with respect to the autonomously replicating sequence (ARS)209 (ARSH4). In the absence of stress, neither wild-type nor *mrc1*^*3A*^ cells showed an increase in TAR. Albeit under control conditions, the *mrc1*^*3A*^ cells already displayed a certain increase in recombination upon stress, and TAR was strongly induced only in *mrc1*^*3A*^ cells when transcription and replication progressed in a head-on orientation (IN) in all stresses tested (Fig. 2a). Therefore, N-terminal phosphorylation of Mrc1 is a key factor for the prevention of TAR upon stress.

We next asked how important it was for the cell to delay S-phase progression and prevent TAR upon stress. We assessed chromosomal instability upon stress in wild-type or *mrc1*^*3A*^ cells using a red-sectoring assay (see “Methods” section). *mrc1*^*3A*^ cells displayed a clear increase in chromosomal instability in response to stress that was not observed in wild-type cells (Fig. 2b). We then monitored Rad52 *foci* to measure recombination events in the genome. Cells containing Rad52-GFP were subjected to osmotic, heat, or oxidative stress and Rad52 *foci* were assessed using microscopy. As expected, stressed *mrc1*^*3A*^ cells, but not stressed wild-type cells, displayed an increase in Rad52 *foci* compared to control (Fig. 2c). These data indicated that N-terminal phosphorylation of Mrc1 is important for maintaining genomic integrity upon stress.

We previously showed that the genomic instability of *mrc1*^*3A*^ cells upon osmostress did not render the cells osmosensitive unless it was in combination with mutations in the DNA damage checkpoint, which prevent the lethal accumulation of the molecular events that are responsible for genomic instability^23^. Similarly, in the present study, deletion of the *MEC1* gene, which encodes a serine threonine kinase that can activate the DNA damage checkpoint, or the *mrc1*^*3A*^ mutation, did not affect cell growth in the presence of other stresses (heat or oxidative). However, its synthetic combination (*mec1 mrc1*^*3A*^) resulted in cells that were heat or oxidative stress sensitive (Fig. 2d). These data suggest that transcription–replication conflicts that occur upon several stresses due to the lack of N-terminal phosphorylation of Mrc1 require the DNA damage checkpoint to maintain cell viability.

### Mutation of *MSN2* and *MSN4* prevents transcription–replication conflicts

**Fig. 3:**
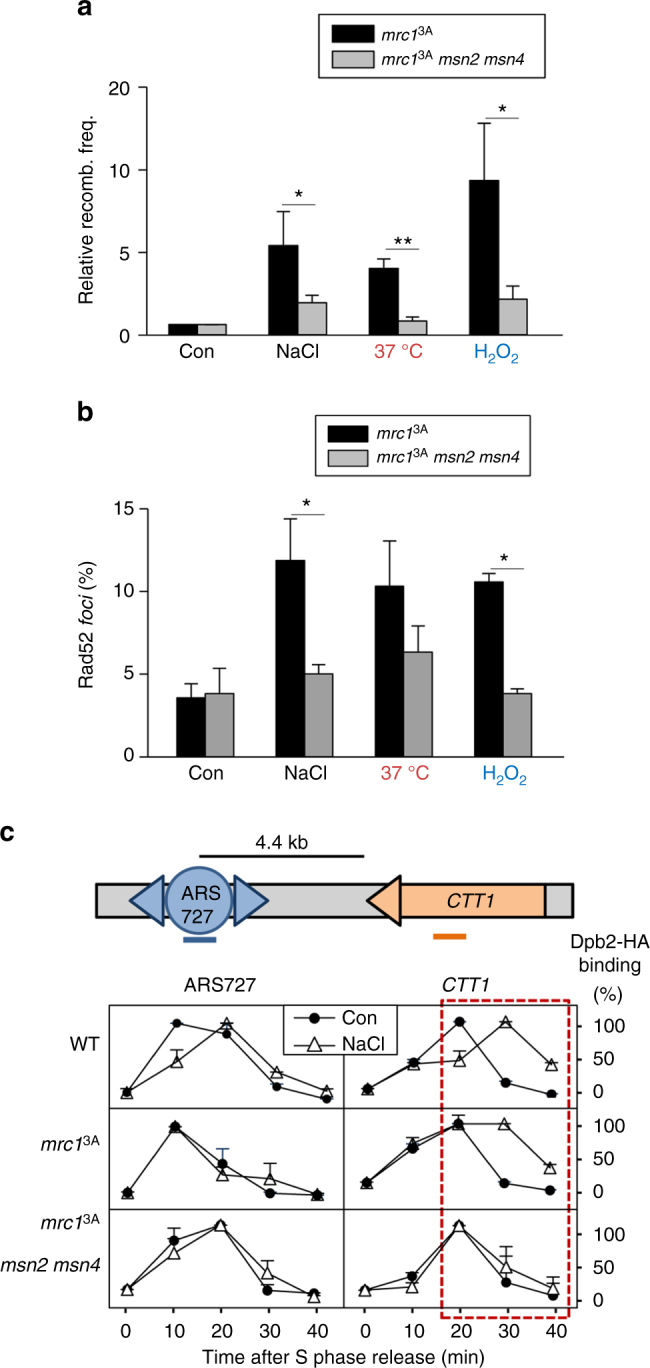
Genomic instability in *mrc1*^3A^ cells depends on transcription. **a** Deletion of the *MSN2* and *MSN4* transcription factors suppresses the high TAR levels of the *mrc1*^3A^ strain upon stress. Numbers are relative to the recombination levels in control conditions. **b** The *mrc1*^3A^*msn2 msn4* strain displays fewer Rad52-YFP *foci* than the *mrc1*^3A^ strain upon stress. Experiments in **a** and **b** were performed as in Fig. 2. Data in **a** and **b** represent the mean and standard deviation of three independent experiments. Values that are significantly different (*p < 0.05, **p < 0.01, ***p < 0.005) by Student's *t* test are annotated. **c** DNA polymerase proceeds more slowly across the *CTT1* gene upon osmostress in *mrc1*^3A^ cells when transcription is active. Schematic diagram of the ARS727 and the *CTT1* loci. The association of Dpb2-HA at the indicated ARS727 and *CTT1* sites (colored bars) was determined using ChIP. Graphs show the kinetics of Dpb2-HA binding after release in YPD (Con) or osmostress (NaCl). The red square highlights the relevant time points. Data represent the mean and standard deviation of three independent experiments

The ESR is mainly mediated by the Msn2 and Msn4 transcription factors^15,28^. Since the expression of the *CTT1* ESR gene is controlled by these transcription factors, we assessed TAR in both *mrc1*^*3A*^ and *mrc1*^*3A*^*msn2 msn4* cells. Induction of TAR in *mrc1*^*3A*^ cells was suppressed by deletion of these transcription factors (Fig. 3a). By assessing Rad52 *foci*, we then investigated if deletion of these transcription factors had a global effect on the recombination events that occur at the genome upon stress. Similar to its inhibition of TAR, the deletion of *MSN2* and *MSN4* also strongly reduced Rad52 *foci* accumulation upon different stresses (Fig. 3b). These data indicated that inhibition of the ESR prevents transcription–replication conflicts upon stress.

To assess conflict between transcription and replication at a genomic locus, we performed ChIP analysis of HA-tagged Dpb2, which is the DNA polymerase epsilon catalytic subunit, to follow the progression of DNA polymerase toward the *CTT1* gene, which is located at a distance of 4.4 kb from the ARS727 early origin. ChIP analyses indicated that, under osmostress conditions, DNA polymerase showed delayed binding to the early origin in wild-type cells when released from S phase that was not observed in the *mrc1*^*3A*^ cells (Fig. 3c). As a consequence of this delay, there was also a delay in DNA polymerase reaching the *CTT1* locus in wild-type cells upon stress compared to *mrc1*^*3A*^ cells. Despite this delay, the residence time of the DNA polymerase on the *CTT1* gene in wild-type cells was similar under the control or the stress condition. In clear contrast, in the *mrc1*^*3A*^ strain, the presence of stress led to an accumulation of the DNA polymerase on *CTT1* that was neither observed in the absence of stress nor in the absence of transcription in the *mrc1*^*3A*^*msn2*Δ *msn4*Δ strain (Fig. 3c). These data show that DNA polymerase encounters problems in proceeding through a highly transcribed genomic locus in *mrc1*^*3A*^ cells, indicating that Mrc1 phosphorylation prevents transcription–replication conflicts in the genome.

### Several kinases phosphorylate the N-terminal region of Mrc1

**Fig. 4:**
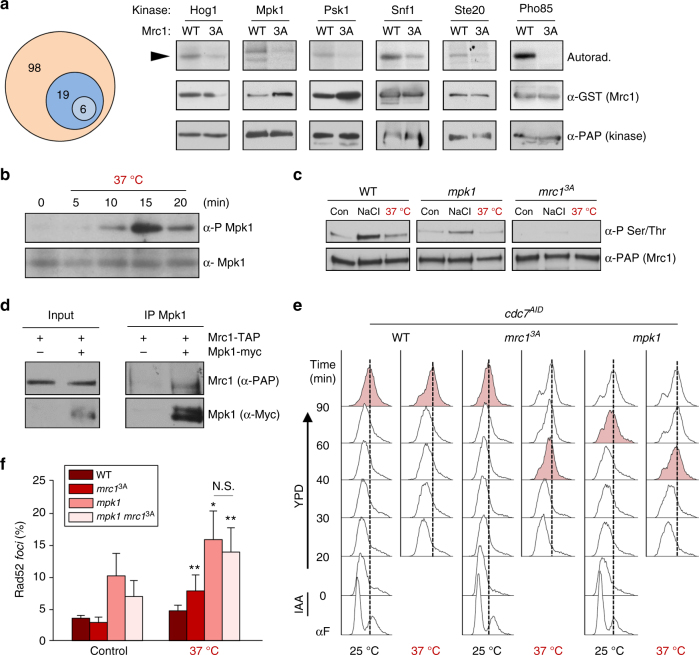
A kinase screen identified several kinases that phosphorylate the N terminus of Mrc1. **a** Six kinases phosphorylate the N terminus of Mrc1. In the screen, 123 tagged-purified yeast kinases were incubated in an in vitro kinase assay with a fragment (1–360 aa) of Mrc1 or its corresponding mutant in which the three phosphorylation sites Thr169, Ser215, and Ser229 were mutated to alanine (*mrc1*^*3A*^) (see “Methods” section). Out of these 123 in vivo-purified kinases, 25 kinases (in blue) phosphorylated GST–Mrc1 in an in vitro kinase assay. Six of these 25 kinases phosphorylated GST–Mrc1 but not GST-*mrc1*^*3A*^. **b**–**f** Mpk1 kinase mediates the Mrc1-dependent cell cycle delay upon heat stress. **b** Mpk1 is phosphorylated in vivo upon heat stress. Synchronized cells were released into S phase and subjected to heat stress. Mpk1 phosphorylation over time was followed by western blotting using specific antibodies. Nonphosphorylated Mpk1 was blotted as a loading control (**c**) Mrc1 is phosphorylated in vivo by Mpk1 upon heat stress. This experiment was carried out as in Fig. 1a and was assayed as in **b**. **d** Mrc1 and Mpk1 interact in vivo. Mrc1–TAP was immunoprecipitated (IP) from cells and coprecipitated Mpk1-myc was assayed using western blotting. **e** *mpk1* cells bypass the DNA replication delay caused by heat stress. Pheromone presynchronized cells were arrested at S-phase onset using the *cdc7*^*AID*^ (auxin-induced degron) system before their release at 37 °C (heat stress). DNA replication over time was followed using FACS. **f** *mpk1* cells display a higher percentage of Rad52-YFP *foci* upon heat stress. Data represent the mean and standard deviation of three independent experiments. Asterisks indicate statistically significant differences by Student's *t* test (*p < 0.05, **p < 0.01, ***p < 0.005) of stress versus control conditions

Hog1 is not activated upon heat or oxidative stress (Supplementary Figure 3A). Additionally, deletion of Hog1 does not alter induction of the ESR (Supplementary Figure 3B), and does not lead to an increase in TAR upon heat or oxidative stress (Supplementary Figure 3C). We therefore hypothesized that kinases other than Hog1 might be responsible for the phosphorylation of the N-terminal region of Mrc1 upon those stresses. To identify those kinases, we performed an unbiased screen in which we tested 123 tagged-purified yeast protein kinases (see “Methods” section) in an in vitro kinase assay using purified GST-tagged Mrc1 or Mrc1^3A^ (amino acids 1–360). This screen yielded 25 kinases that phosphorylated Mrc1, of which only six kinases were able to phosphorylate the wild-type Mrc1 but could not phosphorylate the Mrc1^3A^ mutant (Fig. 4a). One of these six kinases was Hog1, which served as an internal control for the in vitro screen. Mpk1 was identified in this screen as one of the six kinases that can specifically phosphorylate the N-terminal sites of the wild-type Mrc1. Mpk1 is activated in response to high temperatures and it has been implicated in the heat stress response^29^. We therefore tested whether Mpk1 is involved in the regulation of Mrc1 upon heat stress. We assayed Mpk1 phosphorylation (activation) in synchronized cells that were released into S phase and then subjected to heat stress. Mpk1 was rapidly phosphorylated under these in vivo conditions (Fig. 4b and S4A). Heat stress-induced Mrc1 phosphorylation was abolished in both *mrc1*^*3A*^ and *mpk1* mutants, suggesting that Mpk1 phosphorylates the N-terminal region of Mrc1 upon heat stress (Fig. 4c, Supplementary Figure 4A, Supplementary Figure 4B, and Supplementary Figure 4C). We next tested whether Mpk1 and Mrc1 can directly interact by performing immunoprecipitation experiments using cells expressing endogenously Myc-tagged Mpk1 and TAP-tagged Mrc1. TAP-tagged Mrc1 coprecipitated with Myc-tagged Mpk1 (Fig. 4d), indicating that Mrc1 is a bona fide target of Mpk1.

To assess the role of Mpk1 in replication upon heat stress, cells were subjected to heat stress after *cdc7*^*AID*^ synchronization. At 37 °C, the cells replicated faster than at 25 °C. However, cells carrying the *mrc1*^*3A*^ allele or *mpk1* mutation progressed faster than wild-type cells, indicating that Mpk1 delays replication upon heat stress (Fig. 4e). We then followed Rad52 *foci* in these cells under these conditions. Even though the number of *foci* in *mpk1* cells was already higher than that of wild-type cells under nonstress conditions, the number of *foci* further increased in *mpk1* cells upon heat stress. Of note, the synthetic mutation of *mpk1* and *mrc1*^*3A*^ did not lead to a further increase of Rad52 *foci* upon heat stress (Fig. 4f). These data indicate that Mpk1 kinase is responsible for the regulation of Mrc1 to protect genomic stability upon heat stress.

### Psk1 targets Mrc1 to delay S phase upon oxidative stress

**Fig. 5:**
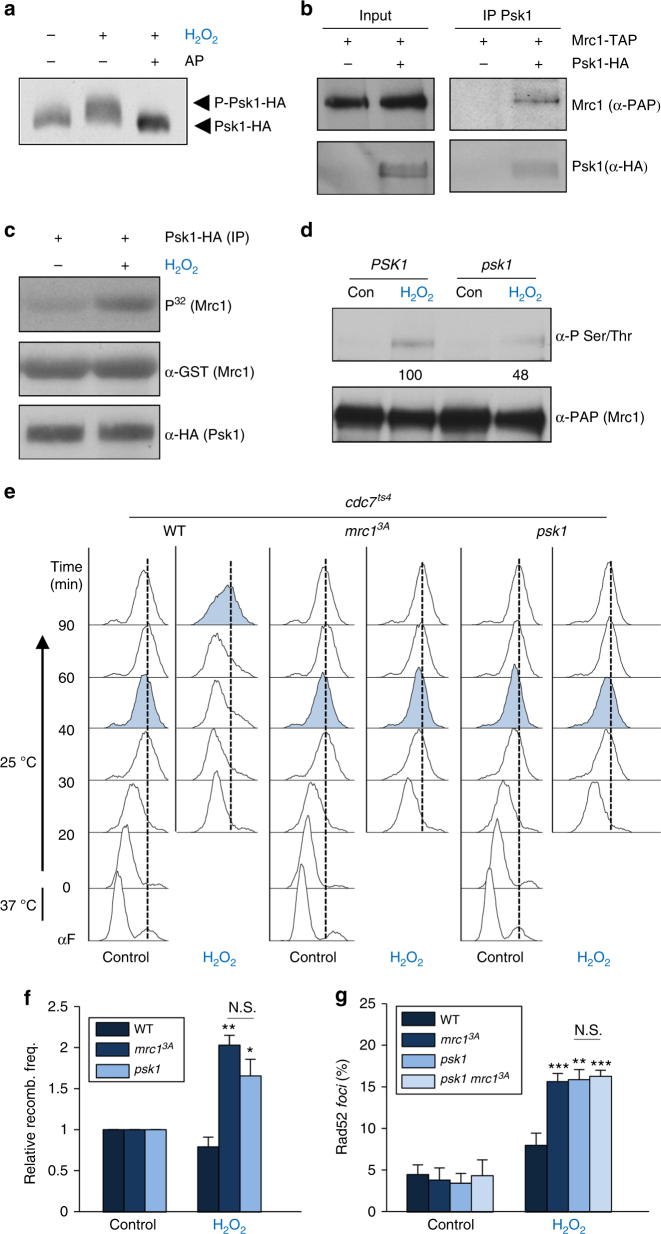
Psk1 mediates the Mrc1-dependent delay upon oxidative stress. **a** Psk1 is phosphorylated upon oxidative stress in vivo. In vitro kinase assay of Psk1–HA immunoprecipitated from treated cells. AP alkaline phosphatase. **b** Mrc1 and Psk1 interact in vivo. Co-immunoprecipitation of Mrc1–TAP with Psk1–HA was assayed in an in vivo pull-down assay and subsequent western blotting with the indicated antibodies. **c** Psk1 phosphorylates Mrc1 more efficiently when activated by oxidative stress. The phosphorylation of GST–Mrc1 by HA–Psk1 immunoprecipitated from treated/untreated cells was assayed using an in vitro kinase assay and autoradiography. **d** Mrc1 is phosphorylated by Psk1 in vivo. This experiment was carried out as in Fig. 1a. Numbers indicate the percentage of phosphorylation relative to loading and the signal of Mrc1 phosphorylation in the wild-type strain. **e** *psk1Δ* cells do not delay S phase upon oxidative stress. DNA replication over time was followed using FACS. **f** *psk1* cells display higher levels of TAR upon oxidative stress than wild-type cells. Numbers are relative to recombination levels in control conditions. **g** *psk1* cells show a higher percentage of Rad52-YFP *foci* upon oxidative stress. Data represent the mean and standard deviation of three independent experiments. Asterisks indicate statistically significant differences by Student's *t* test (*p < 0.05, **p < 0.01, ***p < 0.005) of stress versus control conditions

The involvement of Hog1 and Mpk1 in osmostress and heat stress, respectively, led us to test whether some of the other kinases identified in the screen might promote the delay in S phase observed upon oxidative stress. Since Psk1 was proposed to be involved in the oxidative stress response^30^, we tested whether Psk1 was activated under the oxidative stress conditions that had induced S-phase delay. HA-tagged Psk1 showed a clear shift in mobility in a western blot upon oxidative stress that was abolished by alkaline phosphatase (AP) treatment (Fig. 5a). We then assessed whether Psk1 could interact with Mrc1. HA-tagged Psk1 coprecipitated with TAP-tagged Mrc1, indicating a direct interaction of the two proteins (Fig. 5b). Of note, immunoprecipitated HA–Psk1 phosphorylated purified GST-tagged Mrc1 more efficiently when activated upon oxidative stress (Fig. 5c). Finally, we assessed in vivo Mrc1 phosphorylation in cells upon oxidative stress as before, using wild-type cells or cells deficient in *PSK1*. The phosphorylation of Mrc1 was clearly reduced upon oxidative stress in *psk1* cells in comparison with wild-type cells (Fig. 5d). The combined data indicated that the activity of Psk1 is induced upon oxidative stress and, once activated, Psk1 phosphorylates Mrc1.

Since oxidative stress induces a delay in S phase (Fig. 1), we next asked whether Psk1 was involved in the S-phase delay upon oxidative stress. The delay observed in wild-type cells upon H_2_O_2_ treatment was abolished in both *mrc1*^*3A*^ cells and *psk1* cells, indicating that Psk1 is responsible for the delay in the cell cycle upon oxidative stress (Fig. 5e). We then tested whether Psk1 was relevant for the prevention of TAR upon oxidative stress. Deletion of *PSK1* resulted in an increase in TAR upon oxidative stress similar to that observed in *mrc1*^*3A*^ cells (Fig. 5f). The percentage of Rad52 *foci* also increased upon oxidative stress to a similar extent in *psk1* and *mrc1*^*3A*^ cells. Of note, no additivity was observed in the double mutant *psk1 mrc1*^*3A*^ (Fig. 5g). These data indicated that the Hog1, Mpk1, and Psk1 kinases phosphorylate Mrc1 to delay S phase and to protect genomic integrity upon osmotic, heat, and oxidative stress, respectively.

### Snf1 regulates Mrc1 upon nutrient deprivation

**Fig. 6:**
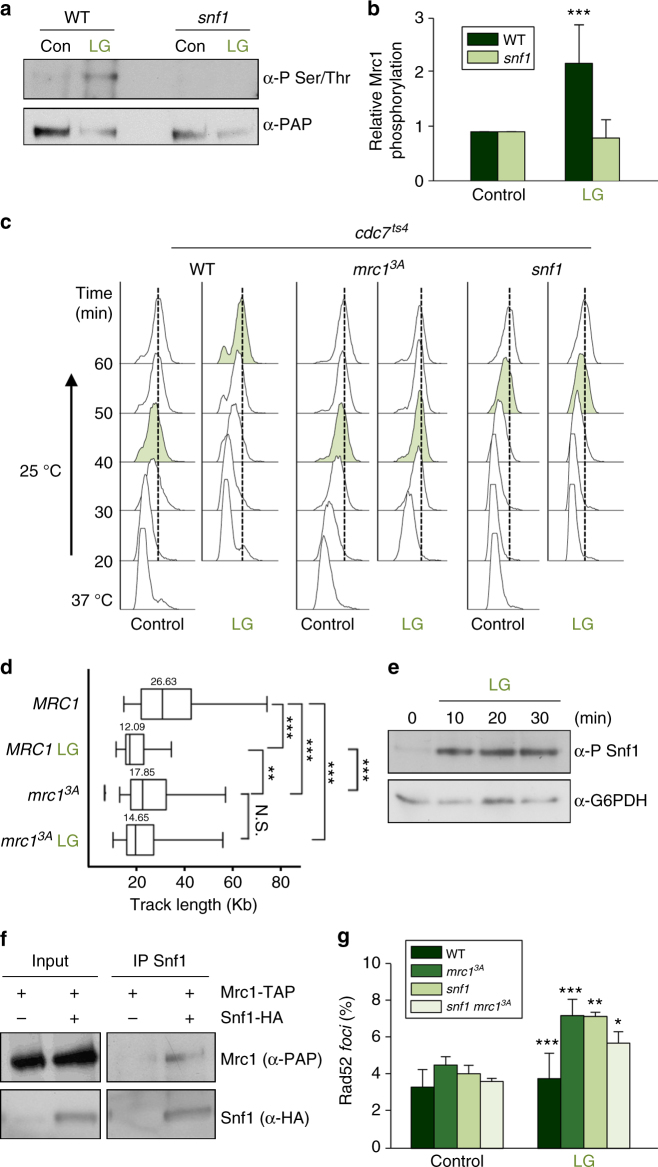
Mrc1 is phosphorylated by Snf1 upon low glucose availability. **a** Mrc1 is phosphorylated by Snf1 in vivo. This experiment was carried out as in Fig. 1a. **b** Quantification of five independent phosphorylation experiments with the indicated strains. Data represent the mean and standard deviation. The graph indicates the relative phosphorylation of Mrc1 (assessed as in **a**) in the indicated strains normalized to the total amount of precipitated Mrc1–TAP protein and then referenced to control conditions. The data represent the mean and standard deviation of five independent experiments. Values that are significantly different (respect to the control conditions) from Student's *t* test are indicated (***p < 0.005). **c** Phosphorylation of Mrc1 by Snf1 delays DNA replication when the cells are subjected to nutrient deprivation (low glucose), whereas *mrc1*^3A^ or *snf1* mutant cells bypass this delay. Cells were synchronized and released in low glucose (LG), and DNA replication over time was followed using FACS. **d** Low glucose delays DNA replication. Replication forks in WT and *mrc1*^*3A*^ cells in control conditions or in LG stress were analyzed using DNA combing. The graph indicates the distribution of BrdU track length (kb) during 1 h of labeling. Box, 25–75 percentile range; Whiskers, 5–95 percentile range. *Values are significantly different (**p < 0.01, ***p < 0.0001). **e** Snf1 is phosphorylated in vivo upon low glucose. Cells were synchronized and released into S phase in low glucose (LG). Snf1 phosphorylation over time was followed using western blotting with specific antibodies. **f** Mrc1 and Snf1 interact in vivo. HA–Snf1 was immunoprecipitated (IP) from cells and coprecipitated Mrc1–TAP was assayed using western blotting. **g** *snf1* and *mrc1*^3A^ cells show a higher percentage of Rad52-YFP *foci* upon low glucose. Data represent the mean and standard deviation of three independent experiments. Asterisks indicate statistically significant differences by Student's *t* test (*p < 0.05, **p < 0.01, ***p < 0.005) of stress versus control conditions

In addition to osmotic, heat, and oxidative stress, it has been reported that low glucose also induces the ESR program^31^. In this respect, a notable finding of the above kinase screen was that Snf1 phosphorylated the N-terminal sites of Mrc1. Mrc1 was phosphorylated in vivo in response to low glucose, and this phosphorylation was abolished in a *snf1* mutant (Fig. 6a, b). Snf1 is the AMP kinase that mediates the cell-adaptive responses to nutrient deprivation^32–34^; however, it has not been previously implicated in the control of S-phase progression. We therefore assessed if Mrc1 might play a role in nutrient deprivation similar to its role in environmental stress. For this purpose, we initially monitored S-phase progression upon low glucose treatment by FACS analysis and monitoring of Clb5 degradation. Low glucose treatment induced a clear delay in cell cycle progression that was almost abolished in *mrc1*^*3A*^ cells (Fig. 6c and Supplementary Figure 5A). This finding indicated that the phosphorylation of Mrc1 is also relevant for the delay in S-phase progression upon low glucose treatment. We then assessed whether low glucose treatment altered DNA replication by performing DNA combing. Wild-type cells showed significantly shorter replication tracks upon low glucose treatment compared to *mrc1*^*3A*^ cells, which indicates a delay in RF progression in the wild-type cells (Fig. 6d). These data indicated that the delay observed in cell cycle progression upon low glucose treatment is caused by a delay in replication mediated by Mrc1 phosphorylation.

To assess the relevance of this delay for cell adaptation to nutrient deprivation, we assayed the effect of low glucose treatment on TAR in wild-type and *mrc1*^*3A*^ cells. The *mrc1*^*3A*^ cells, but not the *MRC1* wild-type cells, showed a clear increase in recombination upon nutrient deprivation (Supplementary Figure 5B). In addition, we also assayed chromosomal instability upon low glucose treatment in wild-type or *mrc1*^*3A*^ cells using a red-sectoring assay. The *mrc1*^*3A*^ cells displayed a clear increase in chromosomal instability (chromosome loss) under low glucose conditions that was not observed in wild-type cells (Supplementary Figure 5C). Furthermore, the synthetic combination of *mec1 mrc1*^*3A*^ resulted in cells sensitive to low glucose (Supplementary Figure 5D). These data indicated that the N-terminal phosphorylation of Mrc1 is critical for the maintenance of genomic integrity under low glucose conditions.

Snf1 is activated by phosphorylation at the Thr210 residue^35^. We followed Snf1 phosphorylation over time under our experimental setup and found that Snf1 was clearly phosphorylated (activated) upon low glucose treatment (Fig. 6e). We then monitored the direct interaction of Snf1 and Mrc1 in vivo by performing immunoprecipitation experiments using cells expressing HA-tagged Snf1 and TAP–Mrc1. HA–Snf1 and TAP–Mrc1 were found to coprecipitate, indicating that they interact in vivo (Fig. 6f). To assess the relevance of the Mrc1 phosphorylation by Snf1 in maintaining genomic integrity, we assayed Rad52 *foci* under low glucose conditions. *mrc1*^*3A*^ and *snf1* cells displayed a clear increase in Rad52 *foci* upon low glucose that did not increase in the double mutant *mrc1*^*3A*^*snf1* (Fig. 6g), indicating that the phosphorylation of Mrc1 by Snf1 protects genomic integrity in low glucose.

### Mrc1 prevents genomic instability in slow growing and unstable cells

**Fig. 7:**
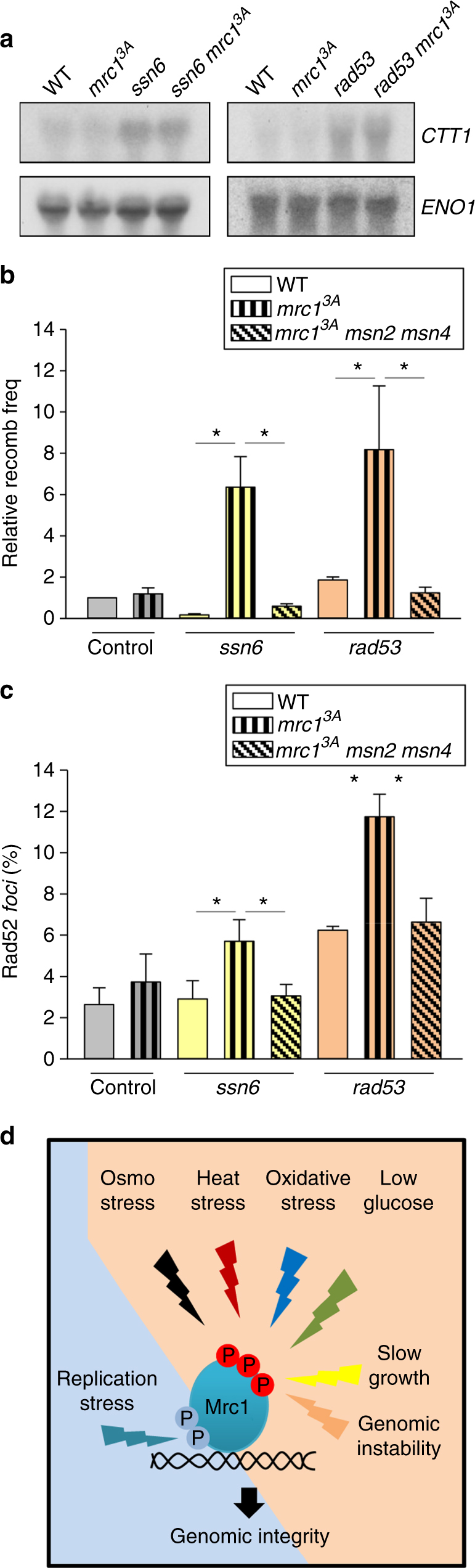
Mrc1 prevents TAR upon slow growth and genomic instability. **a** Slow-growing cells (*ssn6*) and genomically unstable cells (*rad53*) expressed *CTT1* mRNA in the absence of stress. Northern blot analysis of the mRNA expression of *CTT1* in the indicated cells is shown. *ENO1* mRNA expression was assayed as a loading control (**b**) *ssn6* and *rad53* cells display higher levels of TAR when combined with the *mrc1*^3A^ allele. Deletion of the *MSN2* and *MSN4* transcription factors suppresses the high TAR levels of the *ssn6 mrc1*^3A^ and *rad53 mrc1*^3A^ strains. **c** *ssn6* and *rad53* cells show a higher percentage of Rad52-YFP *foci* when combined with the *mrc1*^3A^ allele. Deletion of the transcription factors suppresses the increase in *foci* formation in the *ssn6 mrc1*^3A^ and *rad53 mrc1*^3A^ strains. Data represent the mean and standard deviation of three independent experiments. Values that are significantly different (*p < 0.05, **p < 0.01, ***p < 0.005) by Student's *t* test are annotated. **d** Schematic diagram of the “Mrc1 transcription–replication safeguard mechanism” (MTR). Mrc1 is part of the DNA damage checkpoint that is activated upon replication stress. In addition, the N-terminal phosphorylation of Mrc1 serves to prevent transcription–replication conflicts and to maintain genomic integrity upon transcriptional outbursts during S phase

It has been shown that a number of genetic perturbations that result in slower growth rates also display a common expression signature that resembles the ESR^36^. Similarly, mutations that cause genomic instability also trigger the ESR transcriptional signature^37,38^. We hypothesized that cells carrying mutations that lead to an increased ESR should induce Mrc1 phosphorylation to prevent replication and transcription conflicts during S phase. Initially, using northern blotting, we assessed whether slow-growing cells (i.e., *ssn6*) or cells displaying genomic instability (i.e., *rad53*) expressed the *CTT1* and *ALD3* prototypical ESR genes. Both strains showed higher expression of those genes compared with controls (Fig. 7a and Supplementary Figure 6) consistent with a previous report^36^.

We then assessed whether the mutation of *SSN6* or *RAD53* resulted in an increase in TAR. The single mutation of *SSN6* or *RAD53* genes did not significantly induce TAR compared to wild-type cells. However, in clear contrast, the synthetic mutation of *ssn6* or *rad53* with *mrc1*^*3A*^ resulted in a clear increase in TAR. As expected, these increases in TAR were completely abolished by mutation of the *MSN2* and *MSN4* transcription factors that are responsible for the ESR (Fig. 7b). Additionally, the levels of Rad52 *foci* also clearly increased when *ssn6* or *rad53* mutations were combined with *mrc1*^*3A*^ and these increases in *foci* formation were also abolished by deletion of the *MSN2* and *MSN4* transcription factors (Fig. 7c). These data therefore suggest that Mrc1 function is not restricted to environmental cues but that Mrc1 also integrates internal signals that induce unscheduled changes in transcription that can lead to transcription–replication conflicts (summarized in Fig. 7d).

## Discussion

Transcription–replication conflicts are a major source of genomic instability. Cells have evolved several mechanisms to coordinate the two processes. However, it is not clear how cells deal with unscheduled outbursts of transcription when these occur in S phase. Here, we show that Mrc1 plays a key role in the prevention of transcription–replication conflicts in S phase. In response to transcriptional outbursts, Mrc1 is phosphorylated in its N-terminal region by multiple kinases and this phosphorylation leads to a delayed cell cycle, prevents TAR, and serves to preserve genomic integrity.

In general, transcription is highly coordinated with replication to prevent encounters between the transcription and replication machineries. However, there are some scenarios, such as environmental insults that might occur during S phase, which require a massive induction of gene expression to maximize cell survival. In contrast to the programmed highly active transcription (i.e., of rRNA) that takes place at loci near the specialized regions of the genome (i.e., replication fork barriers) that prevent transcription–replication conflicts, this unscheduled stress-dependent transcription results in the presence of transcription complexes throughout the genome. Thus, in this scenario, it is essential that alternative sophisticated mechanisms exist that can prevent and resolve transcription–replication conflicts. In yeast, the Hog1 SAPK transiently induces hundreds of genes upon osmostress^16,39^. Hog1 also regulates cell cycle progression^40–42^. Within the same temporal framework in which stress-induced transcription occurs during S phase, Hog1 also phosphorylates Mrc1 at its N-terminal region, which prevents the origin of firing and slows down replication fork progression resulting in a delay in S phase^23^. Of note, in contrast to *mrc1*-deficient cells which display replication defects and a delay of S-phase progression under normal conditions, *mrc1*^*3A*^ mutant replicates as the wild type and does not display hallmarks of replication stress. The delay caused by N-terminal phosphorylation upon stress is independent of the DNA damage checkpoint governed by Mec1 and Rad53^22^, and is critical for coordination of osmostress-induced transcription with replication. When cells are deficient in the N-terminal phosphorylation of Mrc1, they display an increase in TAR, genomic instability, and reduced viability upon osmostress. We therefore hypothesized that N-terminal phosphorylation of Mrc1 might represent a general mechanism by which transcription is coordinated with replication upon sudden transcriptional outbursts. Here, we investigated whether replication might be delayed in order to maintain genomic integrity through such a mechanism upon other stresses that do not activate Hog1 but that do induce sudden changes in gene expression to a similar extent as that induced by osmostress. Indeed, multiple environmental stresses such as heat, oxidative stress, and nutrient limitation, which lead to major changes in gene expression by inducing the ESR, did lead to Mrc1 phosphorylation, and this phosphorylation prevented TAR and genomic instability in addition to maximizing cell survival. These results indicated that the N-terminal phosphorylation of Mrc1 serves to integrate multiple signals to control replication and prevent transcription–replication conflicts.

By performing an unbiased biochemical screening, we identified six kinases that phosphorylate the N terminus of Mrc1. Remarkably, most of the kinases identified were signaling kinases, one of which was Hog1. Another of the identified kinases was Mpk1, which is known to be activated upon heat stress^29^. Here, we show that Mpk1 can phosphorylate Mrc1 in response to heat and that this phosphorylation is important for the delay in cell cycle progression and for the prevention of TAR and genomic instability following heat stress. Thus, different MAP kinases, Hog1 in osmostress and Mpk1 in response to heat stress, can phosphorylate the same Mrc1 sites. Little is known regarding Psk1 kinase, although it has been suggested to be involved in oxidative stress^30^. Here, we found that deletion of *PSK1* prevented the cell cycle delay that is induced upon oxidative stress and resulted in an increased TAR and genomic instability upon oxidative stress. We therefore concluded that Psk1 is the kinase that prevents transcription–replication conflicts upon oxidative stress, which defines a new role for this kinase. A notable result of the kinase screen was the identification of Snf1 as a kinase that can phosphorylate Mrc1. Snf1 is the AMP kinase that is responsible for the cellular response to nutrient deprivation and it is highly conserved across eukaryotes^34,43^. These characteristics of the AMP kinase prompted us to investigate whether low glucose resulted in S-phase arrest and to define the role of Snf1 in cell cycle control. Indeed, low glucose treatment resulted in a clear Snf1-mediated S-phase delay that was due to delayed replication. The genomic instability in cells lacking Snf1 was similar to that in cells expressing *mrc1*^*3A*^, indicating that Snf1 is a regulator of S-phase progression. Albeit the kinases identified in the screening are able to target Mrc1 in response to their activation and in response to specific stresses, in contrast to Hog1, they do not control the expression of the ESR genes such as *CTT1* per se (Supplementary Figure 7). The combined data show that Mrc1 integrates signals from several signaling kinases that are activated upon specific stimuli to coordinate gene expression and replication.

The ESR is induced not only upon stress, but also by mutations that result in slow growth^36^ or cause genomic instability^38^. Thus, ESR seems to be a transcriptional signature for a “low fitness” state that can be caused by both external insults and internal genomic alterations. Since N-terminal Mrc1 phosphorylation was clearly required to coordinate ESR induction with replication upon several stresses, we investigated whether Mrc1 phosphorylation also served this purpose in response to mutations that lead to slow growth or genomic instability. Remarkably, mutation of *SSN6*, which results in slow growth^36^, or mutation of *RAD53*, which results in genomic instability^44,45^, did lead to increases in TAR and Rad52 *foci* when combined with *mrc1*^*3A*^. Of note, these effects were totally abolished when Msn2 and Msn4, the transcription factors that control the ESR, were also mutated. Therefore, Mrc1 is essential for the prevention of transcription–replication conflicts and the subsequent genomic instability in multiple situations that compromise cell fitness and trigger induction of the ESR transcriptional defense mechanism.

Based on our results, we propose a model (Fig. 7d) in which Mrc1 integrates multiple signals to delay replication. On the one hand, Mrc1 is part of the DNA damage checkpoint when targeted by Mec1^19,20^. Mec1 phosphorylation of Mrc1 occurs at different sites from those targeted by the signaling kinases identified in the present study. On the other hand, we show that the N-terminal phosphorylation of Mrc1 serves as a key integrator of multiple signals to block DNA replication and prevent transcription–replication conflicts. The role of this N-terminal phosphorylation of Mrc1 goes beyond environmental stresses since it also integrates internal signals triggered by mutations that compromise cellular fitness. We have therefore defined a new cell cycle control mechanism, which we have named the “Mrc1 transcription–replication safeguard mechanism” (MTR), that protects genomic integrity when outbursts of transcription or unscheduled transcription occur during S phase.

## Methods

### Growth media

*E. coli* strains were grown at 37 °C in LB (Luria-Bertani) broth containing 1% bactotryptone, 0.5% yeast extract, and 1% NaCl. Plasmids were amplified using DH5α-competent cells. Yeast proteins were expressed in BL21-competent cells. Each specific strain was grown in LB medium supplemented with ampicillin (100 µg/ml) and/or chloramphenicol (25 µg/ml).

Yeast cells were grown at 25 °C (except for indicated experiments) in yeast extract peptone dextrose (YPD) or synthetic defined (SD) selective media lacking specific amino acids.

### Plasmids

The plasmid pAD88 (*MRC1*) was obtained by cloning the *MRC1* ORF and its promoter (323 nt upstream of the ORF) into the *Bam*HI site of the episomal vector pRS415. The plasmid pAD66 (*mrc1*^*3A*^) was obtained by sequential site-directed mutagenesis of pAD88 phosphorylation sites (Thr169, Ser215, and Ser229) to alanine. The integrative constructs pAD74 (*MRC1*) and pAD75 (*mrc1*^*3A*^) were obtained by *Bam*HI digestion of pAD88 and pAD66, respectively, followed by cloning into the pRS406 integrative vector. Plasmid pAD136 (*mrc1*^*3A*^) contains the *MRC1*^*3A*^ coding region together with the *MRC1* promoter (323 bp) and terminator (400 bp downstream of the STOP codon) inserted into the *Bam*HI site of the integrative vector pRS403. Plasmids pMK152 (3MiniAID) and pNHK53 (os*TIR1*) were used to construct the *cdc7–auxin-inducible degron (AID)* system^46^. Plasmids pAD58 (*MRC1*) and pAD68 (*mrc1*^*3A*^) contain the first 1080 bp of *MRC1* or *mrc1*^*3A*^, respectively, cloned into the *Bam*HI site of the pGEX-6P-1 vector in frame with the *GST* N-terminal tag. Plasmids pAD108 (*CTT1*-IN) and pAD105 (*CTT1*-OUT) are based on the GAL-IN and GAL-OUT vectors, respectively,^9^ and were modified by swapping the *GAL1* promoter with the stress-responsive promoter of the *CTT1* gene.

### Yeast strains

The strains used in this work were derived from W303-1a (*MAT*a*, his3-11,15 leu2-3,112 trp1-1, ura3-1, ade2-1*, and *can1-100*), except for those used for the genomic instability assay or DNA-combing assays, where the YPh277^47^ and E3087 (*MATa, ade2-1, trp1-1, can1-100, leu2-3,112, his3-11,15,* and *GAL ps*i + *RAD5*+) strains were used respectively. The Yeast Kinome Screening was performed using TAP-tagged kinases from a yeast TAP-Collection^48^.

To assess in vivo Mrc1 phosphorylation assays, we used YAD103 (W303-1a *MRC1–TAP*::*KanMX*), YAD162 (W303-1a *mrc1*^*3A*^*-TAP*::*KanMX*), YAD201 (W303-1a *MRC1–TAP*::*KanMX mpk1*::*Hyg*), and YAD275 (W303-1a *MRC1–TAP*::*KanMX psk1*::*Hyg*) strains.

For flow cytometric analyses (FACS), western blotting (of Clb5-HA), and chromatin immunoprecipitation (ChIP) assays, we used the following strains: YAD125 (W303-1a *cdc7-ts4 mrc1*::*KanMX* pAD74::*URA*), YAD126 (W303-1a *cdc7-ts4 mrc1*::*KanMX* pAD75::*URA*), YAD274 (*cdc7-ts4 mrc1*::*KanMX* pAD74::*URA psk1*::*Hyg*), and YAD269 (*cdc7-ts4 mrc1*::*KanMX* pAD74::*URA snf1*::*Hyg*), these strains were used to synchronize cells at the beginning of S phase by using the *cdc7-ts4* thermosensitive system. Strains YBC15 (W303-1a *MRC1–TAP*::*KanMX* pNHK53(*Stu*I)::*URA CDC7-3MiniAID*::*Hygro CLB5-6HA*::*Nat*), YBC16 (W303-1a *mrc1*^*3A*^*-TAP*::*KanMX* pNHK53(StuI)::*URA CDC7-3MiniAID*::*Hygro CLB5-6HA*::*Nat*), and YBC33 (W303-1a *MRC1–TAP*::*KanMX* pNHK53(*Stu*I)::*URA CDC7-3MiniAID*::*Hygro CLB5-6HA*::*Nat mpk1*::*TRP1*) were used to synchronize cells using the cdc7-*AID* system. *mpk1* cells were grown in the presence of an osmoprotectant to prevent cellular lysis.

Strains based on YAD125 and YAD126 were used for DNA-combing experiments upon oxidative stress and glucose starvation. These strains were modified to allow incorporation of exogenous BrdU into genomic DNA. Five copies of the herpes simplex *TK* gene under the control of the yeast GDP promoter were inserted at the *URA3* locus to construct YAD292 (E3087 *URA3*::*URA3/GPD-TK(5*×) *AUR1c::ADH-hENT1*) and YAD303 (YAD292 *mrc1*::*Hyg* pAD136::*HIS*). For DNA-combing experiments upon heat stress, the E3087 and yAD303 strains were modified to be synchronized using the *cdc7-AID* system: YBC40 (E3087 *URA::HIS* pNHK53(*Stu*I)::*URA cdc7-MiniAID*::*Hyg*) and YBC45 (YAD303 *URA*::*LEU* pNHK53(*Stu*I)::*URA cdc7-MiniAID*::*Hyg*).

The following strains were used to perform recombination assays, microscopy experiments, and northern blot analyses: YAD103, YAD162, and YAD273 (W303-1a *mrc1*^*3A*^*-TAP*::*KanMX msn4*::*Nat msn2*::*Hyg*), YAD201, YAD200 (W303-1a *mrc1*^*3A*^*-TAP*::*KanMX mpk1::Hyg*), YAD275, YBC1 (W303-1a *mrc1*^*3A*^*-TAP*::*KanMX psk1*::*Hyg*), YAD263 (W303-1a *MRC1–TAP*::*KanMX snf1::Hyg*), YAD306 (W303-1a *mrc1*^*3A*^*-TAP*::*KanMX snf1*::*Hyg*), YAD293 (W303-1a *MRC1–TAP*::*KanMX ssn6*::*Nat*), YAD294 (W303-1a *mrc1*^*3A*^*-TAP*::*KanMX ssn6*::*Nat*), and YAY185 (W303-1a *hog1*::*KanMX*). To perform recombination assays, the corresponding strains were transformed with either pAD105 or pAD108. To perform microscopy experiments to detect Rad52-YPF *foci*, the corresponding strains were transformed with the pWJ1344 plasmid.

To perform red-sectoring assays, the YAD146 strain (yPH277 *mrc1*::*KanMX*) transformed with either pAD88 or pAD66 was used.

To assess protein interactions, the following strains were used: YAD103, YAD211 (W303-1a *MPK1*–*MYC*::*Hyg*), YAD218 (YAD103 *MPK1–MYC*::*Hyg*), YAD285 (W303-1a *SNF1*-*6HA*::*Hyg*), YBC7 (YAD103 *SNF1-6HA*::*Hyg*), YAD284 (W303-1a *PSK1*-*6HA*::*Hyg*), and YBC8 (YAD103 *PSK1*-*6HA*::*Hyg*). In all cases, tags (–TAP, –MYC, and –HA) were integrated at the protein C terminus.

To assess growth, the following strains were used: YAD103, YAD162, YAD117 (W303-1a *sml1 mec1*::*URA*), YAD243 (YAD117 *mrc1*::*KanMX* pAD136::*HIS*), YAD6, and YBC2 strains.

### Synchronization and stress conditions

Synchronization of cells at the onset of S phase for FACS, DNA-combing experiments, and chromatin immunoprecipitation (ChIP) assays was performed in two steps. Overnight cultures were diluted at OD_660_ = 0.3 and grown for 2 h at 25 °C in YPD. In the first step, the cells were incubated with α-factor for 2 h at 25 °C (40 µg/ml) to presynchronize them in G1. In the second step, the cells were washed and incubated in preheated media at 37 °C for 2 h (for *cdc7*^*ts4*^ cells) or were washed and incubated in media containing 5 mM indole acetic acid (IAA) (for *cdc7*^*AID*^ cells) at 25 °C for 2 h. The cells were then released from the S block to progress into S phase at 25 °C, in YPD or in the presence of stress. Synchronization of cells at the onset of S phase for recombination assays, red-sectoring assays, or microscopy experiments was performed in a single step. Cells were synchronized at late G1 with α-factor (40 µg/ml) for 2 h at 25 °C, were washed, and were then released at 25 °C for 35 min before being subjected to the corresponding stress. Except for the indicated experiments, the cells were subjected to the following stress conditions: oxidative stress (0.1 mM H_2_O_2_), heat stress (37 °C), osmostress (0.4 M NaCl), and glucose deprivation (YP––0.05% glucose).

### Immunoprecipitation assays

The selected strains were grown to mid-log exponential phase (OD_660_ = 1) and were left unstressed or were stressed as described above except for oxidative stress where cells were stressed in 2 mM H_2_O_2_. The cells were then collected (400 ml per condition) and kept at −80 °C. Cell pellets were resuspended in 2 ml of lysis buffer (45 mM Hepes-KOH at pH 7.2, 150 mM NaCl, 1 mM EDTA, 10% glycerol, and 1% Triton X-100) containing a cocktail of protease and phosphatase inhibitors. An equal volume of glass beads (0.5-mm diameter) was added, and the cells were broken by vortexing at 4 °C. The whole extract was clarified by centrifugation for 10 min at 9300 × *g* at 4 °C and an aliquot was taken as the whole-cell extract (WCE). The extracts (3–7 mg) were first incubated for 3 h with 1:100 dilutions of anti-Myc antibody (9E10) or with anti-HA antibody (12CA5) and were subsequently incubated overnight with the protein G affinity matrix (GE Healthcare). For purification of the TAP-tagged proteins, cell extracts were directly incubated with rabbit IgG–Agarose (Sigma) overnight at 4 °C. The agarose beads were then washed 10 times with the lysis buffer. Antibody-bound fractions and the corresponding WCE were boiled in SDS-containing sample buffer and were analyzed using 8% SDS-PAGE. TAP-tagged proteins were detected with a 1:5 dilution of anti-PAP antibody (p1291 from Sigma).

### Western blotting

TCA protein extracts were resolved using SDS-PAGE and were then blotted onto a PDVF membrane. Following incubation of the blots with the indicated antibodies, signals were detected using the ECL detection reagent (Amersham). For protein quantification, the corresponding films were scanned using 16 bits/channel and quantified using Quantity One Analysis Software 4.6.1 (BioRad). For resolving Mrc1–TAP mobility shift, 10 µM of phostag was added to the 6% SDS-PAGE gels (37.5:1 acrylamide vs. bisacrylamide).

### Flow cytometric analysis

For flow cytometric analysis, cells were synchronized at S-phase onset and were released in control conditions or in the presence of stress as described above. Cells were fixed in 70% ethanol and were then treated overnight with 1 mg/ml RNAse A at 37 °C in 50 mM sodium citrate. Cells were stained with propidium iodide at a final concentration of 4 µg/ml in 50 mM sodium citrate and were analyzed using a FACSCalibur (BD). A total of 10,000 cells were analyzed for each time point using WinMDI 2.9 Software.

### DNA combing

Mid-log-phase cells were synchronized as described above and were then pulse-labeled with BrdU (Sigma) after their release into S phase in the presence (or not) of the specific stress. DNA combing was performed as described^49^. In panel G, cells were synchronized with alpha-factor and then released at 37° to synchronize them in S phase given that the strain is cdc7ts; when H_2_O_2_ was added, then cells were incubated at 25°. In contrast, in panel H, cells were not cdc7ts. Instead, they carry a Cdc7 auxin-inducible degron allele, and thus, they were synchronized with alpha-factor and synchronized in S phase by IAA at 25°. Then, they were released at 25° or 37° neither with IAA nor with alpha-factor. DNA fibers were extracted in agarose plugs after BrdU labeling and were stretched on silanized coverslips. The DNA fibers were counterstained with anti-ssDNA antibody (DSHB) and a goat anti-mouse antibody coupled to Alexa 546 (A11030, Molecular Probes). BrdU was detected with the BU1/75 (AbCys) anti-BrdU antibody and a goat anti-rat antibody coupled to Alexa 488 (A11006, Molecular Probes). DNA fibers were analyzed using a Leica DM6000 microscope equipped with a DFC390 camera (Leica). Data acquisition was performed with LAS AF (Leica). Even though in panel H we cannot exclude the possibility of fused replicons, we still observed that replication in *mrc1*3A is faster at 37° either because of faster replication forks or activation of origins, indicating that indeed there was an altered DNA replication.

### Recombination assays

Selected strains were transformed with either pAD108 (*CTT1*-IN) or pAD105 (*CTT1*-OUT) vectors (which contain the origin of replication ARSH4 as indicated in Fig. 2a). Cells were grown up to OD_660_ = 0.4 and synchronized as described. Cells were released into S phase and left for 3 h in a control condition or in the presence of stress, following which 200 µl of the cells were plated in SD Trp^−^ plates (control plates, 1/500 dilution) or in SD Trp^−^ Leu^−^ plates (recombination plates, 1/50 dilution). The final recombination levels were calculated as the ratio of recombinant colonies (colonies in SD Trp^−^ Leu^−^ plates) versus the total number of colonies (colonies in SD Trp^−^ plates).

### Red-sectoring plasmid instability assays

Overnight cultures of YAD146 transformed with the plasmid pAD88 or pAD66 were diluted to an OD_660_ = 0.3, grown in YPD until an OD_660_ = 0.5, and synchronized with α-factor (40 µg/ml) for 90 min at 25 °C. Cells were washed and released into S phase in YPD for 30 min and incubated (or not as control) for 3 h in the presence of stress. Cells (200 µl; diluted 1/1000) of each culture were plated in YPD plates and incubated at 30 °C for 3 days and were then stored at 4 °C for about 5 days. The final percentage of cells with plasmid loss was calculated as the percentage of red colonies among the total number of colonies (red and white colonies)^47^.

### Rad52-YFP *foci* assay

Rad52-YFP *foci* were assessed in synchronized cells bearing the plasmid pWJ1344^50^. Cells were incubated in the presence of stress conditions for 2 h after 35-min release from the synchronization with α-factor, and the percentage of cells containing Rad52-YFP *foci* was quantified using a Nikon 90i microscope.

### Expression and purification of recombinant proteins

*E. coli* cells were grown at 37 °C to an OD_600_ = 0.5. GST-tagged proteins were then induced for 6 h by adding 1 mM IPTG at 25 °C. After induction, the cells were collected by centrifugation and resuspended in 1/50th the volume of STET 1× buffer (100 mM NaCl, 10 mM Tris-HCl at pH 8.0, 10 mM EDTA at pH 8.0, and 5% Triton X-100) supplemented with 2 mM DTT, 1 mM PMSF, 1 mM benzamidine, 200 μg/ml leupeptin, and 200 μg/ml pepstatin. Ice-cold cells were lysed by a brief sonication and the lysate was cleared by high-speed centrifugation. GST-fused proteins were pulled down from the supernatants with 300 μl of 4B gluthatione–sepharose beads (GE Healthcare, 50% slurry equilibrated with STET 1×) by mixing them for 90 min at 4 °C. The gluthatione–sepharose beads were collected by a brief centrifugation and were washed four times in STET 1× buffer and twice in 50 mM Tris-HCl buffer at pH 8.0 supplemented with 2 mM DTT. The GST-fused proteins were then eluted in 200 μl of 50 mM Tris-HCl buffer at pH 9.5 supplemented with 2 mM DTT and 10 mM reduced glutathione (Sigma) by rotating for 30 min at 4 °C, and were stored at −80 °C. Wild-type Mrc1 and Mrc13A proteins, either full length or a peptide comprising the first 360 amino acids, were purified from pAD57, pAD58, pAD67, or pAD68, respectively.

### Yeast kinome screening

In total, 123 TAP-tagged Ser/Thr kinases were immunoprecipitated from yeast (50 ml of exponentially growing cultures) as described above (see Immunoprecipitation Assays). Aliquots (30 µl) of each kinase bound to IgG beads were divided into two pools that were used to separately assay phosphorylation of an N-terminal fragment (360 amino acids) corresponding to wild-type or Mrc13A mutant recombinant proteins. The kinase assay was performed as follows: 1 μg of GST-*MRC1* or GST-*mrc1*^*3A*^ that was previously purified from *E. coli* was incubated with the IgG bead-bound kinase in 1× kinase buffer (50 mM Tris-HCl at pH 7.5, 10 mM MgCl_2_, and 2 mM DTT) supplemented with 100 μM cold ATP and 5 µM γ-^32^P ATP. The mixture was incubated for 30 min at 30 °C and each kinase assay was resolved by 8% SDS-PAGE. Proteins were visualized by western blotting and the phosphorylation was detected using Kodak Biomax XAR films (Sigma-Aldrich).

### Growth curves

Yeast cultures were grown to saturation overnight and diluted to OD_660_ = 0.05 the next morning. Triplicates of the indicated strains were grown in sterile 96-well plates under the indicated stress conditions for 10–15 h and the OD_660_ was monitored every hour with the Synergy H1 Hybrid Reader (Biotek).

### Northern blotting

Yeast cultures were grown to mid-log phase in YPD and were kept under control conditions or were stressed for 30 min under the stress conditions as detailed above (see Stress conditions). Total RNA was extracted, resolved in 1% formaldehyde-containing agarose gels, and transferred to nylon membranes. Transcription of the indicated genes was probed using labeled PCR fragments (High Prime DNA Labeling Kit; Roche). Autoradiographic images were obtained using Kodak Biomax XAR films (Sigma-Aldrich) or a Phosphorimager.

### Chromatin immunoprecipitation (ChIP) assay

In ChIP experiments, cells were treated as above, but were released into S phase at 16 °C to follow the kinetic association of Dpb2-HA with DNA sequences. Cells (50 ml per time point) were collected at OD_660_ = 0.7 and were treated with 1% formaldehyde for 20 min at room temperature. Glycine (330 mM) was then added for 15 min. Cells were collected, were washed four times with ice-cold TBS (20 mM Tris-HCl, pH 7.5, 150 mM NaCl), and were then kept at −20 °C for further processing. Cell pellets were resuspended in 0.3 ml of cold lysis buffer (50 mM HEPES-KOH, pH 7.5, 150 mM NaCl, 1 mM EDTA, 1% Triton X-100, 0.1% sodium deoxycholate, 0.1% SDS, and 1 mM PMSF). An equal volume of glass beads (0.5 mm in diameter) was added and the cells were disrupted using Vortex Gene for 13 min on ice. The lysate was diluted into 0.6 ml of lysis buffer and the glass beads were discarded. The cross-linked chromatin was sonicated to yield an average DNA fragment size of 350 base pairs (bp) (range, 100–850 bp). Finally, the sample was clarified by centrifugation at 16,100 × *g* for 5 min at 4 °C. The chromatin solution (600 µl) was incubated with 50 µl of anti-HA monoclonal antibody precoupled to anti-mouse IgG-conjugated paramagnetic beads (Dynabeads M-450; Dynal). After rotation for 90 min at 4 °C, the beads were washed twice for 4 min in 1 ml of lysis buffer, twice in 1 ml of lysis buffer with 500 mM NaCl, twice in 1 ml of washing buffer (10 mM Tris-HCl, pH 8.0, 0.25 M LiCl, 1 mM EDTA, 0.5% N-P40, and 0.5% sodium deoxycholate), and then once in 1 ml of TE (10 mM Tris-HCl, pH 8.0, 1 mM EDTA). Immunoprecipitated material was eluted twice from the beads by heating for 10 min at 65 °C in 50 µl of elution buffer (25 mM Tris-HCl, pH 7.5, 10 mM EDTA, and 0.5% SDS). To reverse cross-linking, samples were adjusted to 0.3 ml with elution buffer and were incubated overnight at 65 °C. After extraction with phenol–chloroform–isoamyl alcohol and chloroform, DNA was ethanol-precipitated for 4 h at −20 °C in the presence of 20 µg of glycogen and was resuspended in 30 µl of TE buffer. For real-time PCR, oligonucleotides for the *ARS727* origin region (ARS727Fwd: GTTCTACTTTAAATGTAGTCAG and ARS727Rev: GAACTGTTCAATACATCAGC) and for *CTT1* gene regions (CTT1Fwd: GGAACAAGACCAAATCAGAAACG and CTT1Rev: CTTTGATCTTACAAGCGTGG) were used.

### Quantification and statistical analysis

Error bars in bar graphs represent the standard deviation (SD) of three independent experiments. Experiments were performed in triplicate using three biological replicates.

Statistical analysis was performed by using the Student's *t* test except for the DNA-combing experiments in which statistical analyses were performed using the Mann–Whitney *U* test, where *p* values indicated with asterisks were considered significantly different (*p < 0.05, **p < 0.01).

The intensity of the bands in western blots was quantified using ImageJ v1.47 (http://imagej.nih.gov/ij).

### Data availability

Flow cytometry was performed using the BD FACSCalibur™ cytometer controlled by the BD™ Worklist Manager Software. WinMDi v2.9 software was used to analyze the flow cytometric data.

DNA fibers were analyzed using a Leica DM6000 microscope equipped with a DFC390 camera (Leica). Data acquisition was performed with LAS AF (Leica).

Rad52-YFP fluorescent *foci* were followed using a Nikon 90i microscope controlled by MetaMorph v7.1.2.0 (Molecular Devices).

Growth curves were constructed using the Synergy H1 Multi-Mode Reader from BioTek controlled by Gen5™ v 2.01.14 Software.

ChIP data were analyzed using the ViiA™ 7 Real-Time PCR System controlled by the ViiA 7 RUO Software v1.2 (Applied Biosystems).

All data that support the findings of this study are available from the corresponding authors on request.

## Electronic supplementary material

Supplementary Information
