# Supplementary material for: Multiple signaling kinases target Mrc1 to prevent genomic instability triggered by transcription-replication conflicts
Source: Nat Commun. 2018 Jan 25;9:379. doi: 10.1038/s41467-017-02756-x (PMC5785523; doi:10.1038/s41467-017-02756-x)
Supplement: Supplementary file 1 — Supplementary Information [file 41467_2017_2756_MOESM1_ESM.pdf]

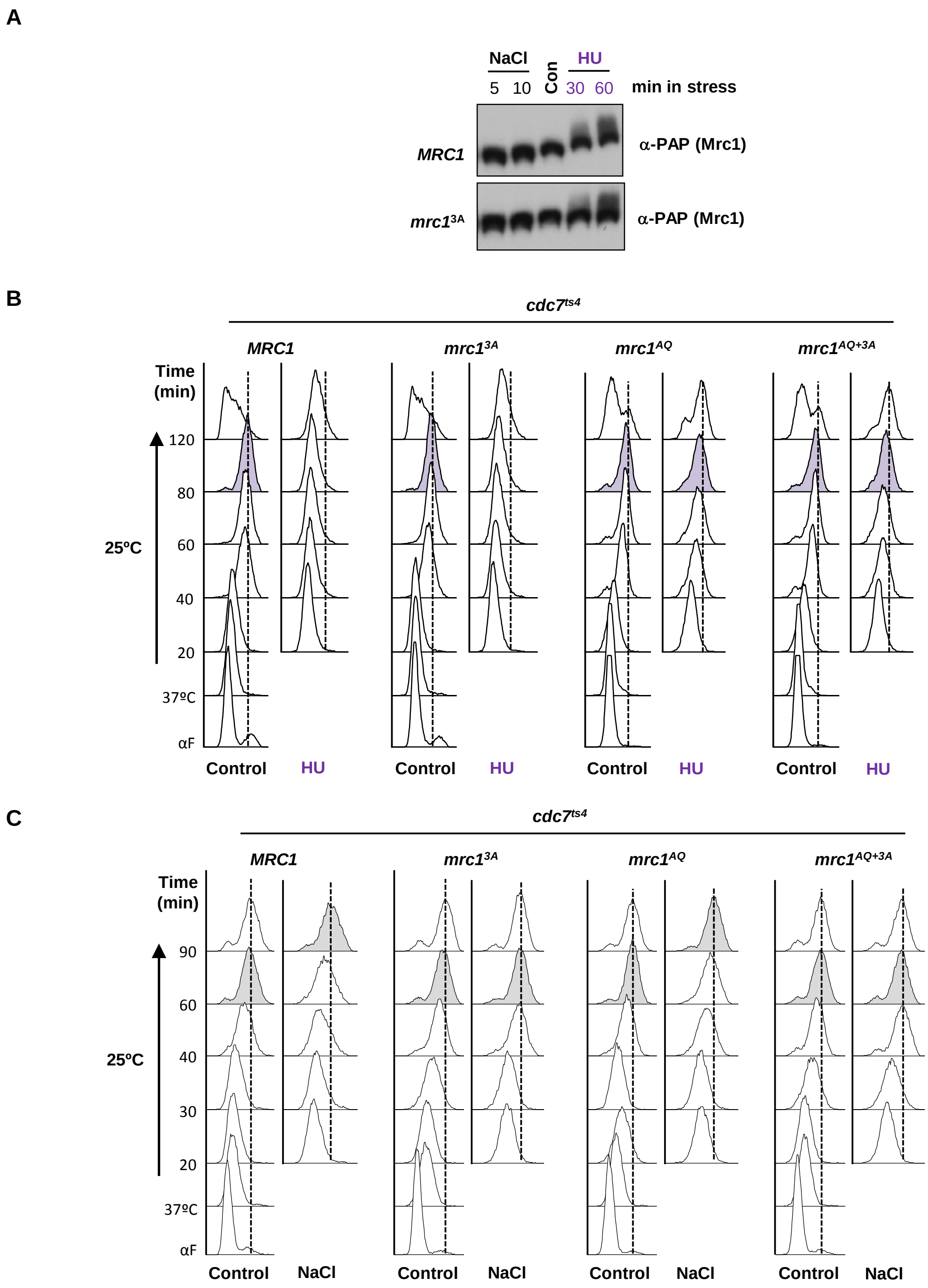

Supplementary Figure 1. *mrc1^AQ* and *mrc1^3A* alleles have genetically separable functions. **(A)** Mrc1 phosphorylation upon 0.2M HU induces an electrophoretic mobility shift that can be detected in phostag-containing gels (see Methods), while environmental stresses like NaCl don't provoke changes in Mrc1 mobility. **(B,C)** *mrc1^AQ* (with all putative Mec1 sites mutated to Ala) and *mrc1^3A* strains were synchronized as described (see Methods) and released upon replicative stress (0.05M HU, in D) or osmostress (0.4M NaCl, in E) and replication progression was followed by FACS.

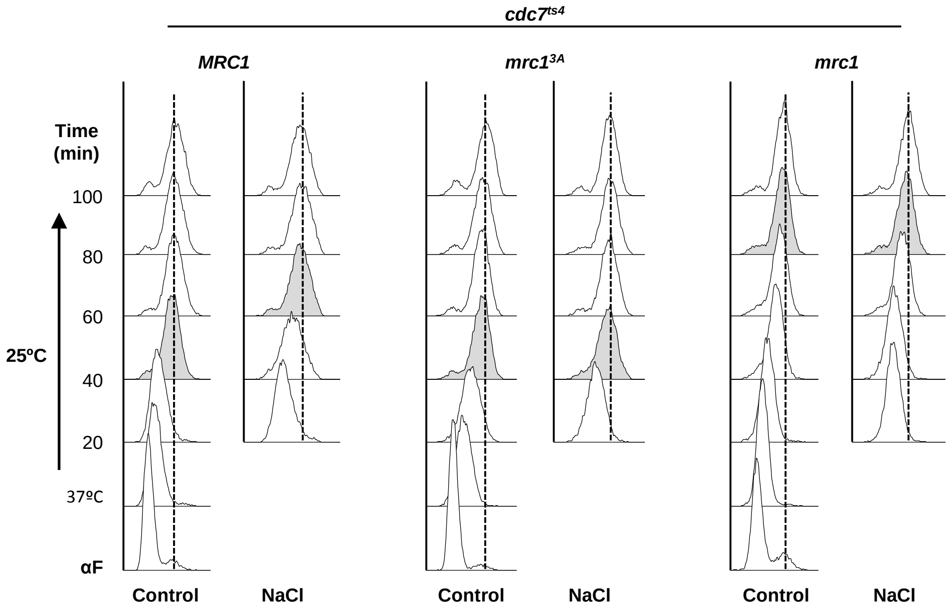

Supplementary Figure 2. *mrc1* shows a delay in DNA replication under control conditions when compared to *MRC1* and *mrc1*^{3A} cells. The indicated strains were synchronized as described (see Methods) and released in control conditions or in osmostress (0.4M NaCl) and replication progression was followed by FACS.

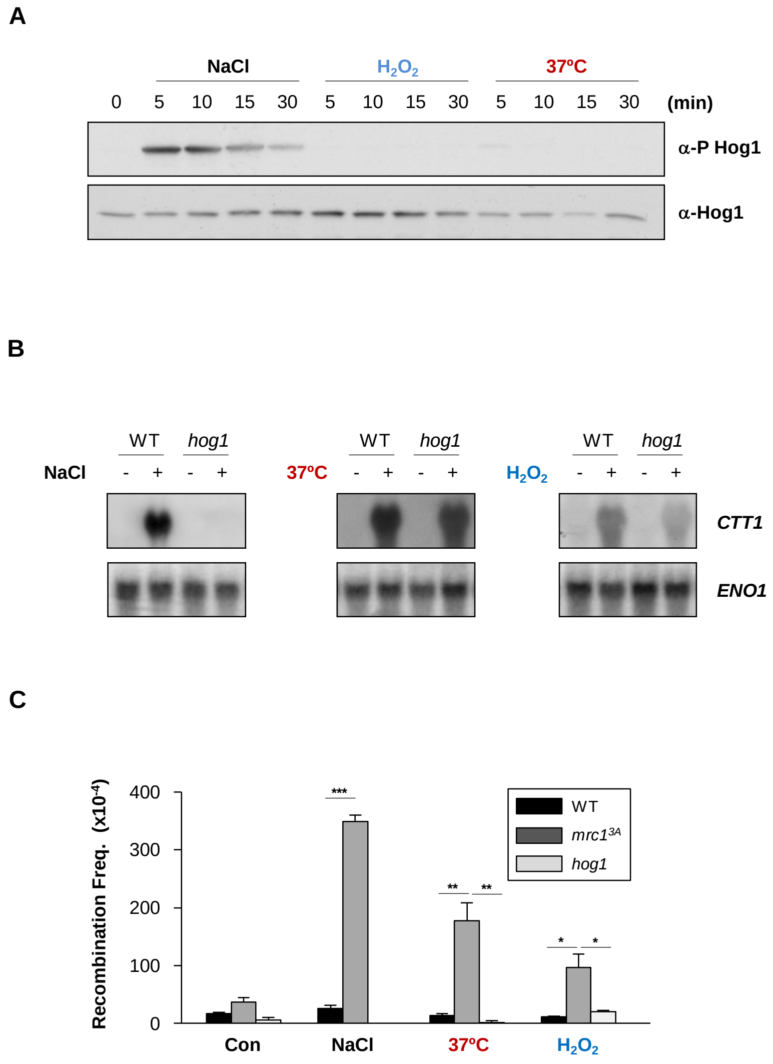

Supplementary Figure 3. Hog1 does not mediate the regulation of Mrc1 upon heat and oxidative stresses. (A) Hog1 is not phosphorylated upon oxidative and heat stresses. Western blotting of phosphorylated and total Hog1 protein is shown. **(B)** Hog1 is not required for induction of *CTT1* expression upon heat or oxidative stress. *CTT1* expression was analyzed in wild type (WT) and *hog1* cells upon the indicated stress conditions using Northern blotting. *ENO1* was blotted as a control. **(C)** *mrc1*^{3A} but not *hog1* cells display associated recombination (TAR) upon oxidative and heat stresses. TAR was not determined in *hog1* cells upon osmostress due to their osmosensitivity. Data represent the mean and standard deviation of three independent experiments. Values significantly different (*p<0.05, **p<0.01, ***p<0.005) by t-student test are annotated.

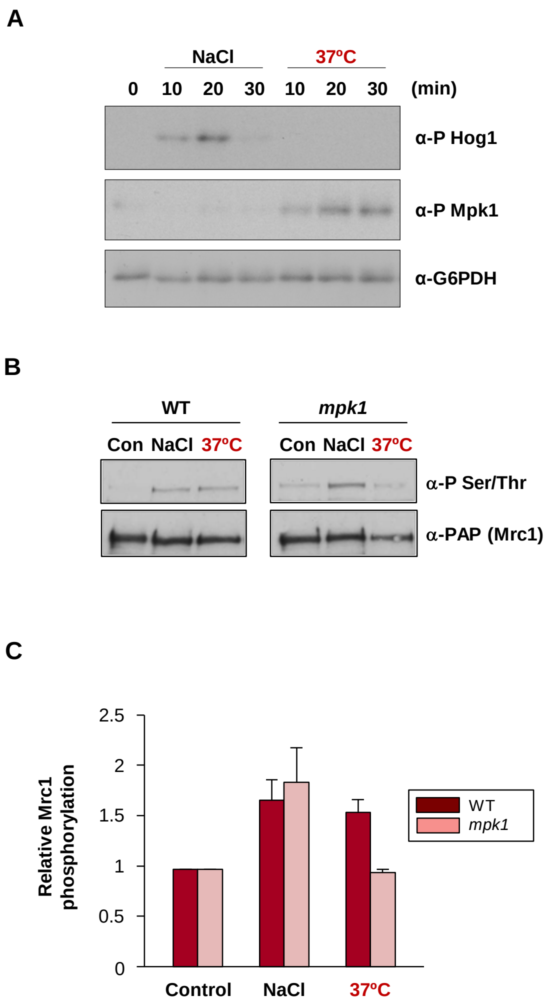

Supplementary Figure 4. Mpk1 phosphorylates Mrc1 upon heat stress. **(A)** Hog1 and Mpk1 are specifically activated upon osmostress or heat stress respectively. Hog1 phosphorylation or Mpk1 phosphorylation was assessed using specific phospho-antibody. G6PDH was monitored as a loading control. **(B)** Mrc1 is phosphorylated *in vivo* by Mpk1 upon heat stress but not upon osmostress. Mrc1 phosphorylation over time was followed by western blotting as in Figure 1A. **(C)** Quantification of phosphorylation experiments with the indicated strains. Data represent mean and standard deviation.

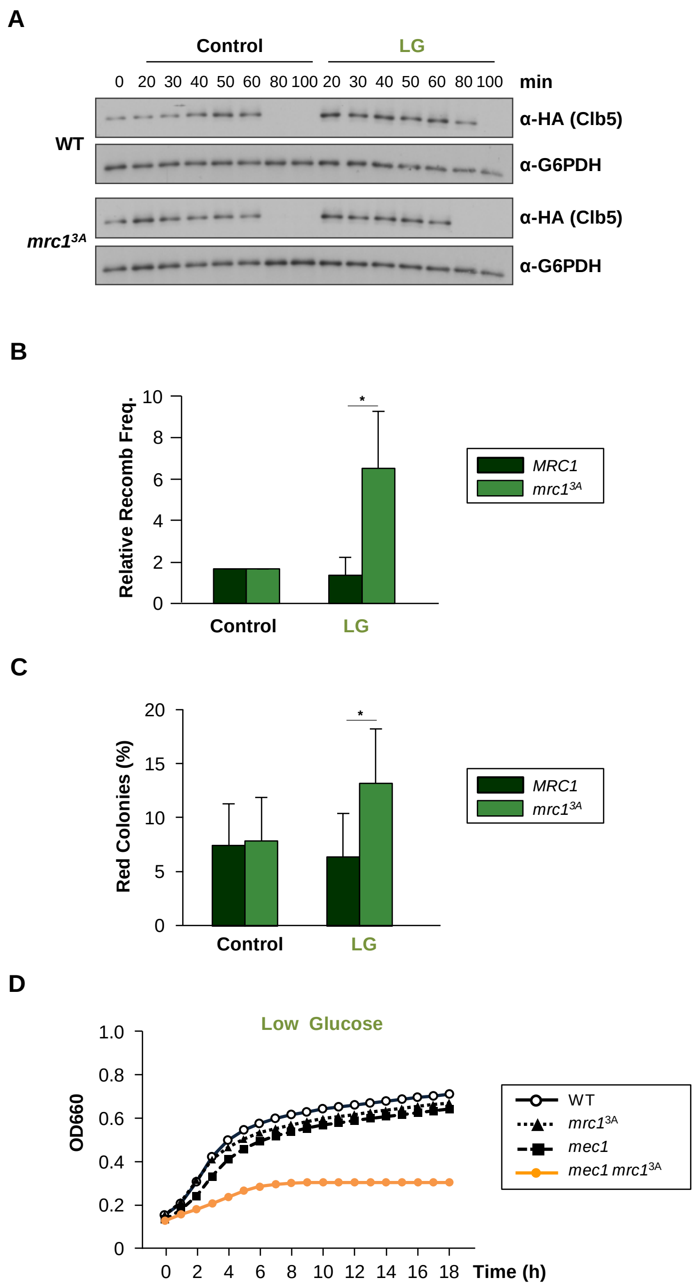

Supplementary Figure 5. N-terminal Mrc1 phosphorylation is required for cell cycle delay and to prevent TAR and genomic instability in response to nutrient deprivation (low glucose).(A) Wild type cells delay Clb5 degradation when subjected to low glucose in contrast to *mrc1^{3A}* cells. Cells were synchronized at the beginning of S phase as described (see STAR methods) and released into S phase at 25 ºC in YPD (Control) or in low glucose (YP-0.05% Dextrose)(LG). Clb5 degradation was assessed using western blotting. **(B)** *mrc1^{3A}* cells display higher levels of recombination than wild type cells in TAR assays upon LG stress (see STAR methods). **(C)** *mrc1^{3A}* cells show higher frequency of plasmid loss upon LG stress in a red sectoring assay (see STAR methods). Data in (B) and (C) represent the mean and standard deviation of three independent experiments. Values significantly different (*p<0.05, **p<0.01, ***p<0.005) by t-student test are annotated. **(D)** *mrc1^{3A}* cells are sensitive to LG when the DNA damage checkpoint pathway is impaired. The indicated strains were grown to log phase and incubated in LG.

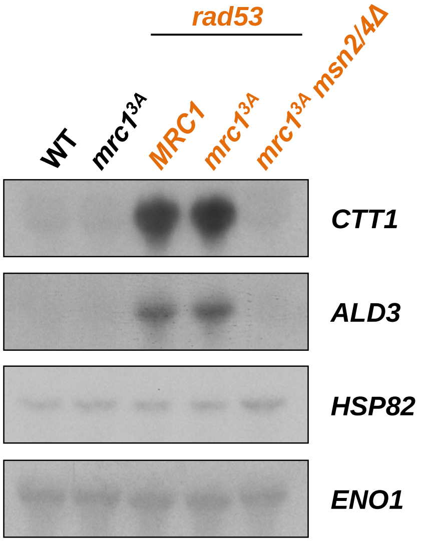

Supplementary Figure 6. Mutation of the *RAD53* gene triggers the canonical ESR response. *rad53* cells (wild type or containing the *mrc1*^{3A} mutation) trigger the transcription of *CTT1* and *ALD3* ESR genes in a Msn2 and Msn4-dependent manner. Northern blot analysis is shown. *HSP82* and *ENO1* are loading controls.

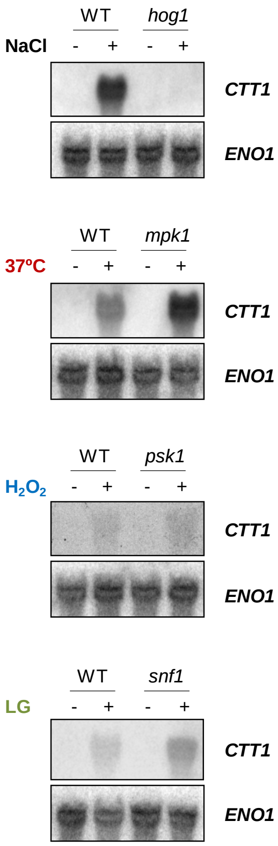

Supplementary Figure 7. Mpk1, Psk1 and Snf1 are dispensable for the expression of the prototypical ESR gene *CTT1* upon heat, oxidative and low glucose stress. *CTT1* expression was analyzed by Northern Blot in wild type (WT) and *mpk1*, *psk1 and snf1* cells upon the indicated stresses. *ENO1* was assessed as a control of total RNA levels.

1A

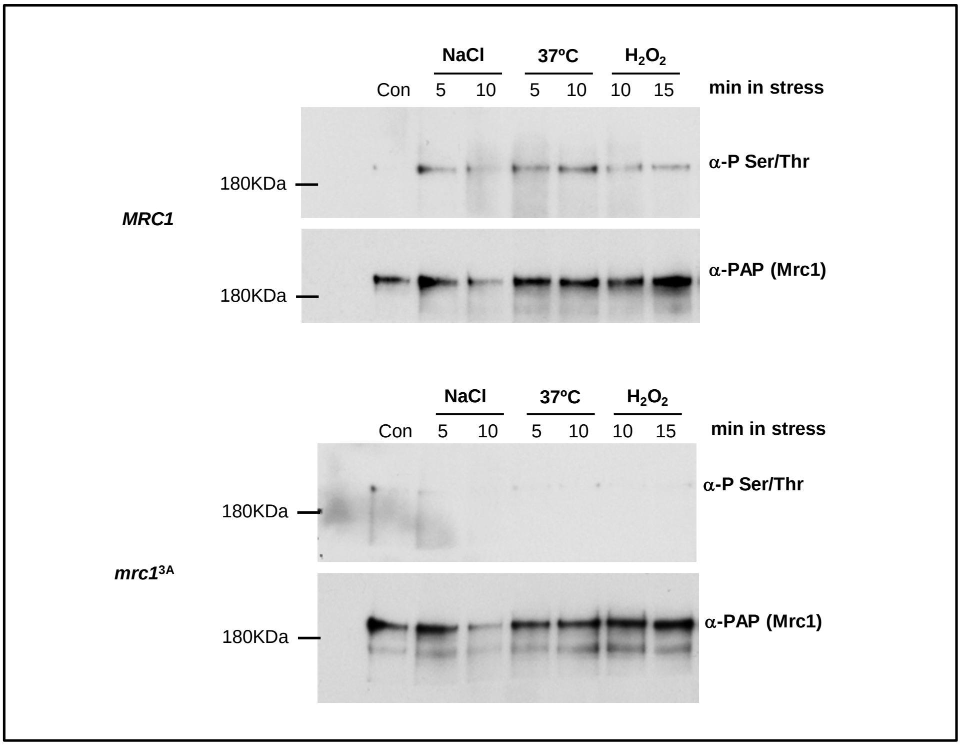
NaCl
37ºC
H_2O_2
Con 5 10 5 10 10 15 min in stress
180KDa
α-P Ser/Thr
MRC1
180KDa
α-PAP (Mrc1)
NaCl
37ºC
H_2O_2
Con 5 10 5 10 10 15 min in stress
180KDa
α-P Ser/Thr
mrc1[3A]
180KDa
α-PAP (Mrc1)

1E

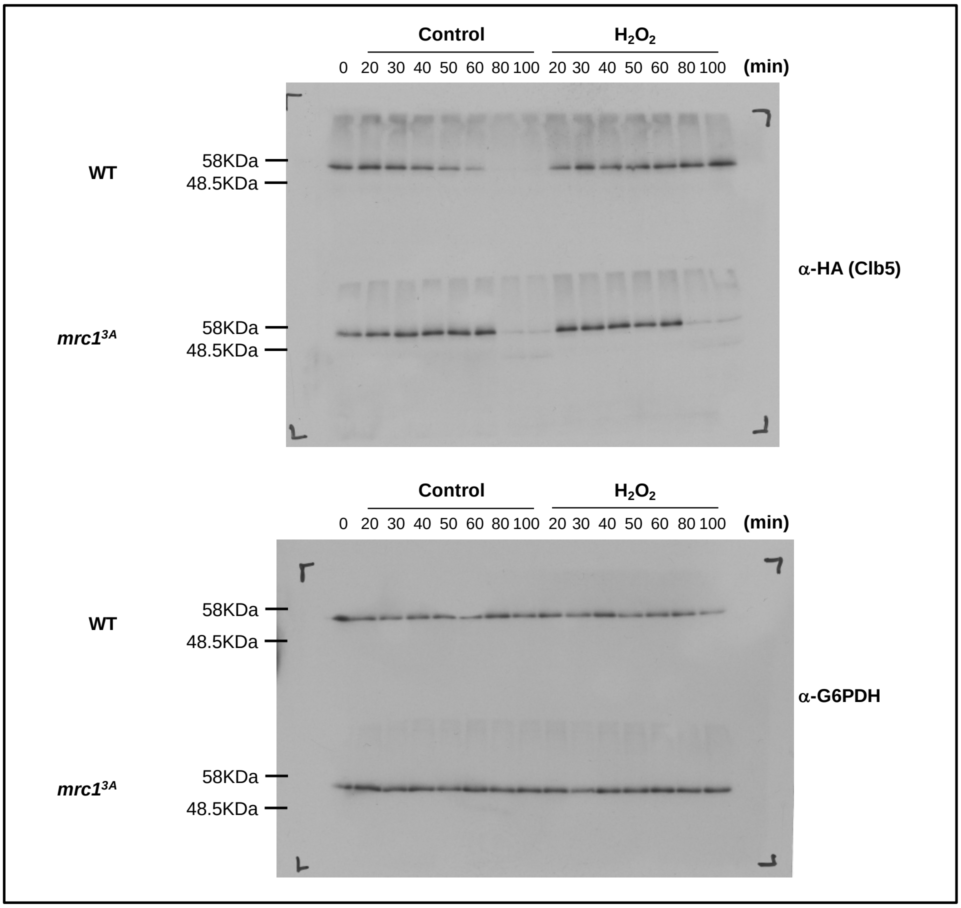
Control
H_2O_2
0 20 30 40 50 60 80 100 20 30 40 50 60 80 100 (min)
WT
58KDa
48.5KDa
α-HA (Clb5)
mrc1[3A]
58KDa
48.5KDa
Control
H_2O_2
0 20 30 40 50 60 80 100 20 30 40 50 60 80 100 (min)
WT
58KDa
48.5KDa
α-G6PDH
mrc1[3A]
58KDa
48.5KDa

1F

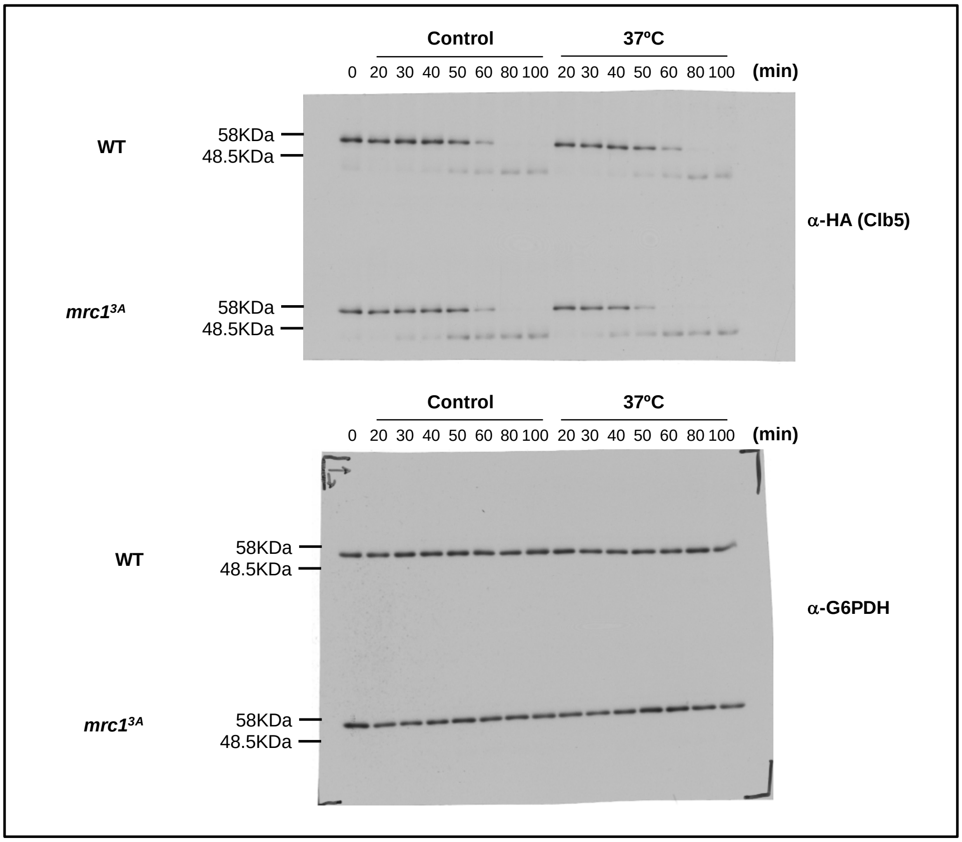
Control
37ºC
0 20 30 40 50 60 80 100 20 30 40 50 60 80 100 (min)
WT
58KDa
48.5KDa
mrc1^3A
58KDa
48.5KDa
α-HA (Clb5)
Control
37ºC
0 20 30 40 50 60 80 100 20 30 40 50 60 80 100 (min)
WT
58KDa
48.5KDa
mrc1^3A
58KDa
48.5KDa
α-G6PDH

4A

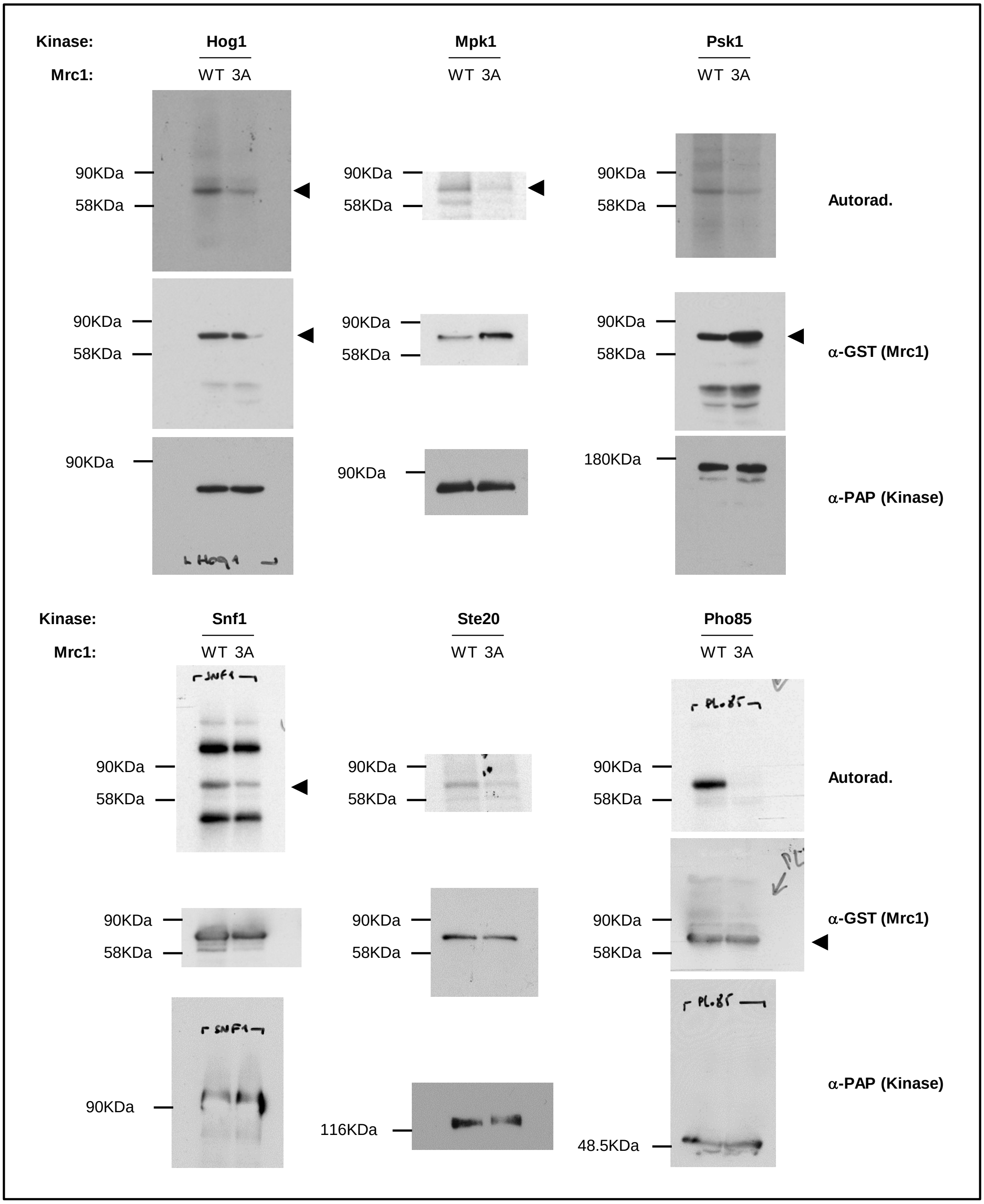

Kinase:
Hog1
Mpk1
Psk1
Mrc1:
WT 3A
WT 3A
WT 3A
90KDa
58KDa
Autorad.
α-GST (Mrc1)
α-PAP (Kinase)
180KDa
Kinase:
Snf1
Ste20
Pho85
Mrc1:
WT 3A
WT 3A
WT 3A
116KDa
48.5KDa

4B

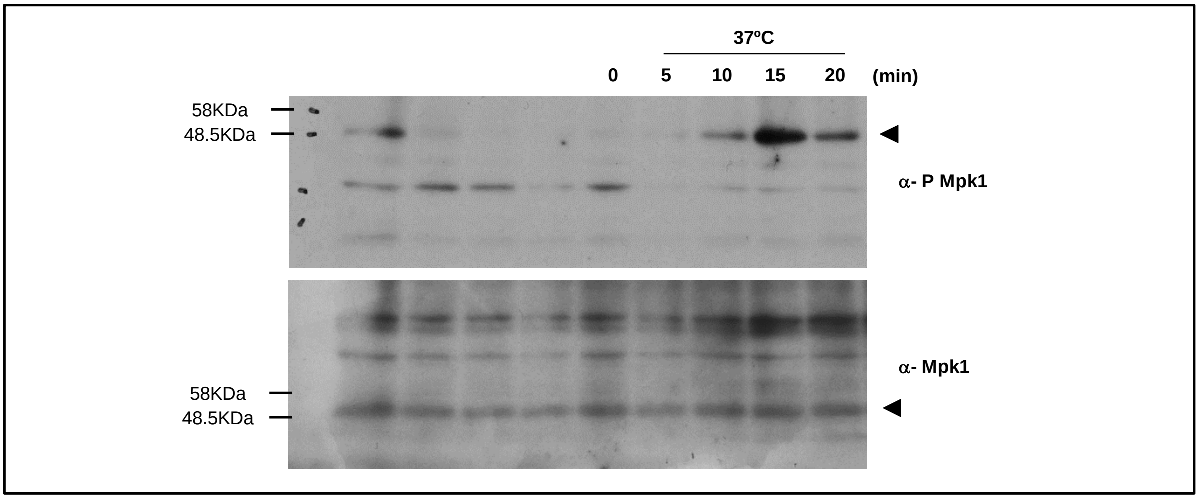
37ºC
0
5
10
15
20
(min)
58KDa
48.5KDa
α- P Mpk1
α- Mpk1
58KDa
48.5KDa

4C

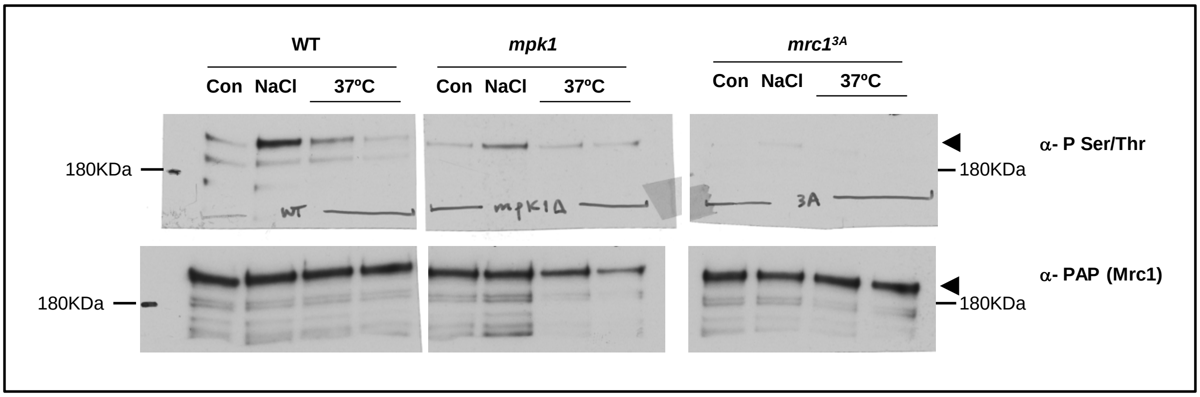
WT
mpk1
mrc1^3A
Con
NaCl
37ºC
α- P Ser/Thr
180KDa
α- PAP (Mrc1)
180KDa

4D

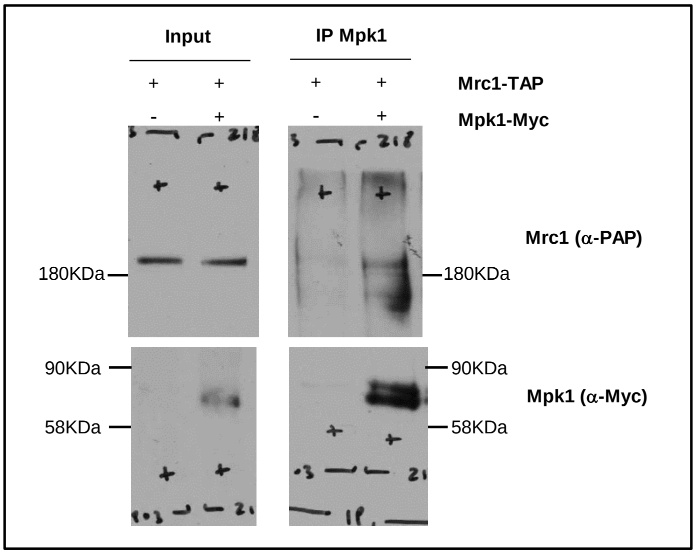
Input
IP Mpk1
Mrc1-TAP
Mpk1-Myc
Mrc1 (α-PAP)
180KDa
90KDa
58KDa
Mpk1 (α-Myc)

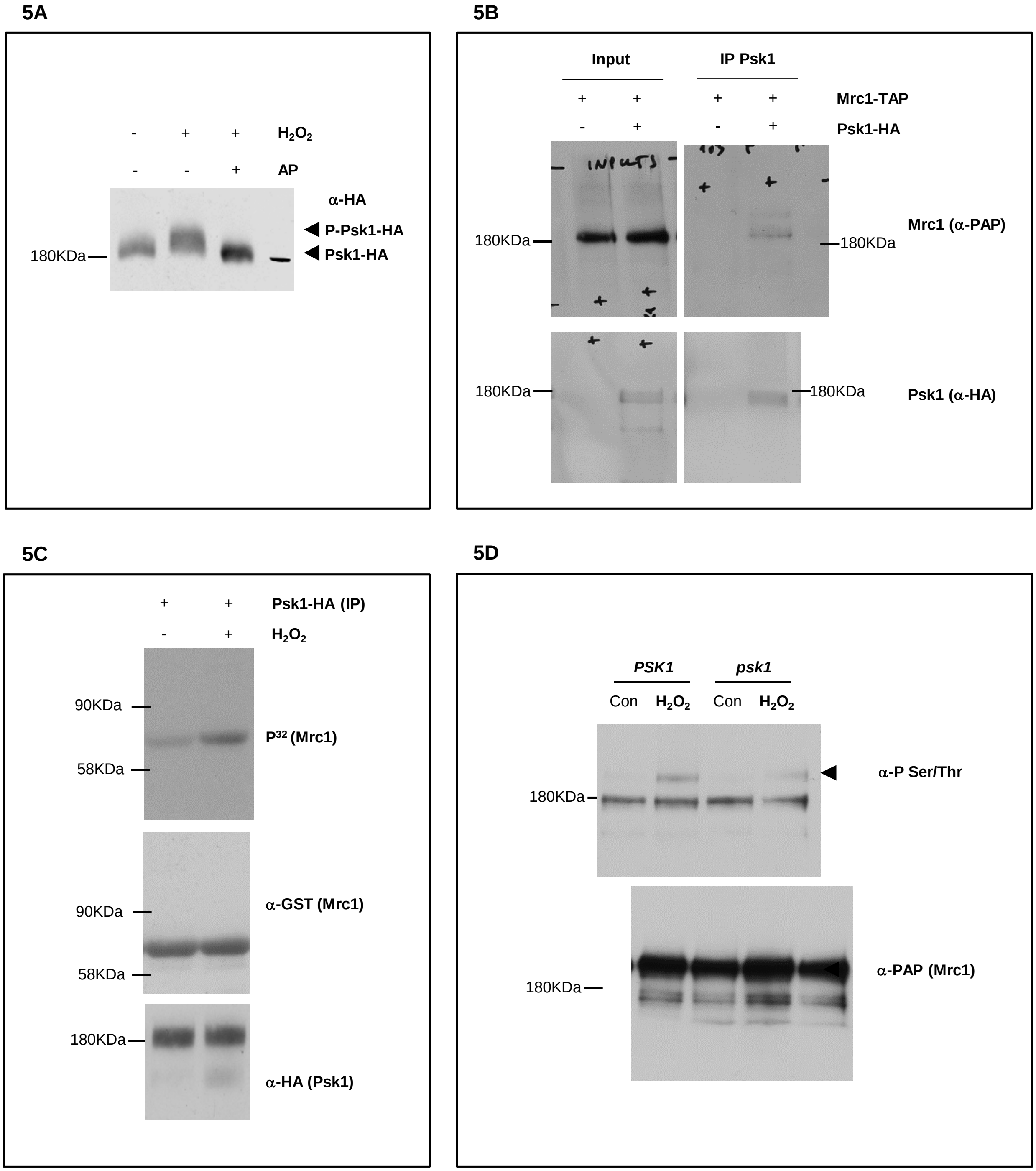
5A
- + + H2O2
- - + AP
α-HA
P-Psk1-HA
Psk1-HA
180KDa
5B
Input
IP Psk1
+ + + + Mrc1-TAP
- + - + Psk1-HA
180KDa
180KDa
Mrc1 (α-PAP)
180KDa
180KDa
Psk1 (α-HA)
5C
+ + Psk1-HA (IP)
- + H2O2
90KDa
58KDa
P32 (Mrc1)
90KDa
58KDa
α-GST (Mrc1)
180KDa
α-HA (Psk1)
5D
PSK1
psk1
Con H2O2 Con H2O2
180KDa
α-P Ser/Thr
180KDa
α-PAP (Mrc1)

6A

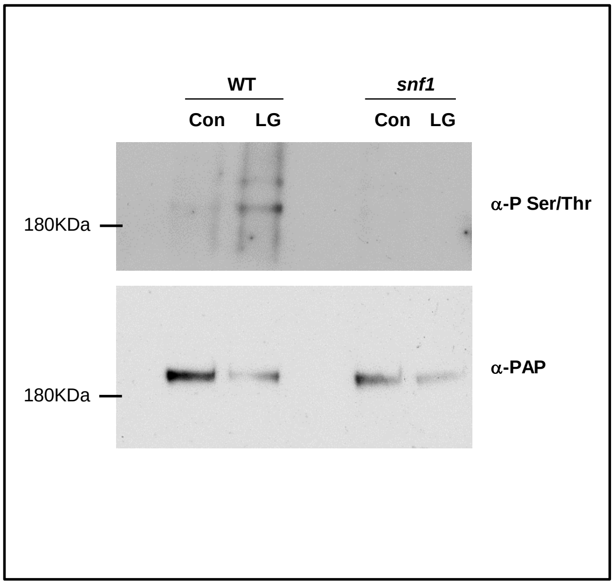
WT
snf1
Con LG Con LG
180KDa
α-P Ser/Thr
α-PAP
180KDa

6E

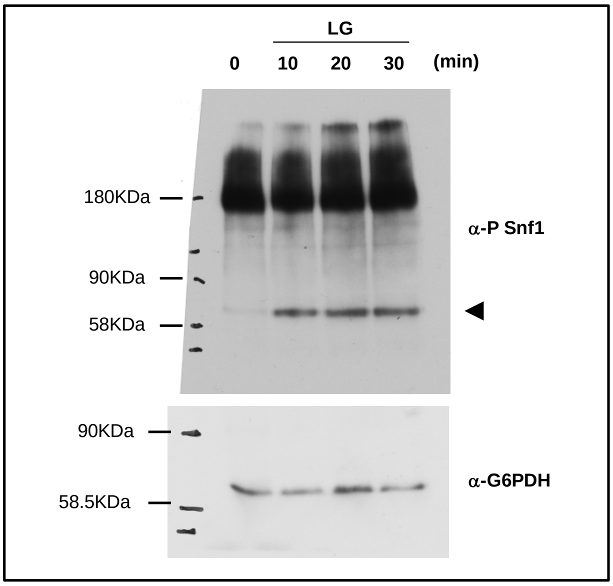
LG
0 10 20 30 (min)
180KDa
90KDa
58KDa
α-P Snf1
90KDa
58.5KDa
α-G6PDH

6F

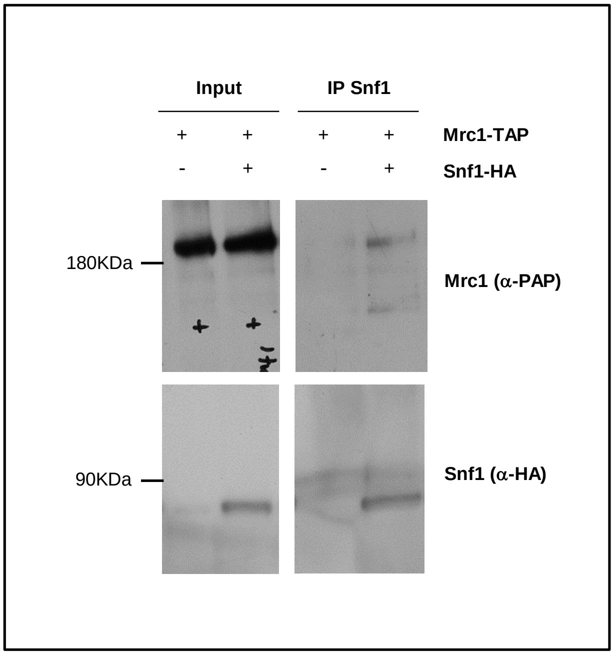
Input
IP Snf1
+ + + + Mrc1-TAP
- + - + Snf1-HA
180KDa
Mrc1 (α-PAP)
90KDa
Snf1 (α-HA)

S1A

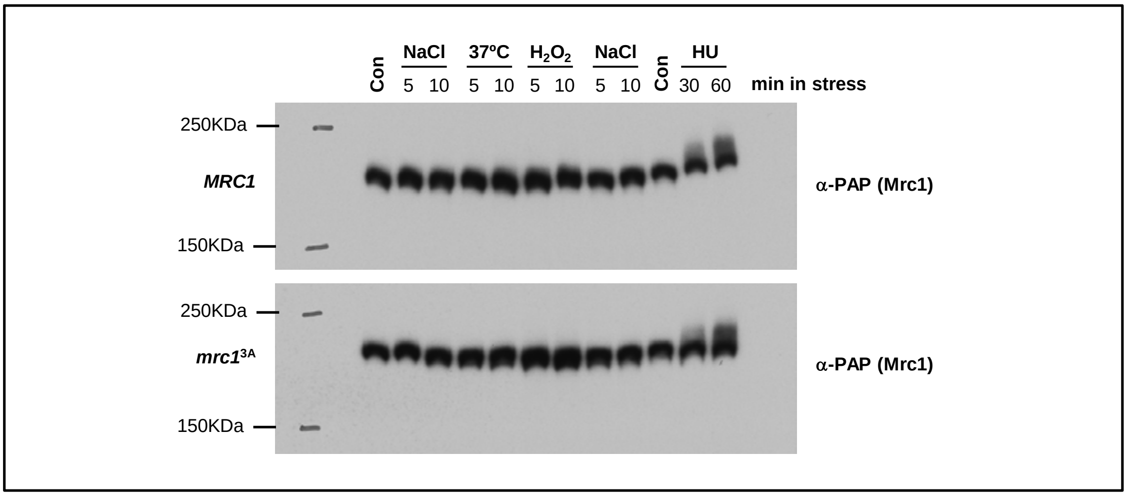

Con
NaCl
5 10
37ºC
5 10
H2O2
5 10
NaCl
5 10
Con
HU
30 60
min in stress
250KDa
MRC1
150KDa
α-PAP (Mrc1)
250KDa
mrc1 3A
150KDa
α-PAP (Mrc1)

S3A

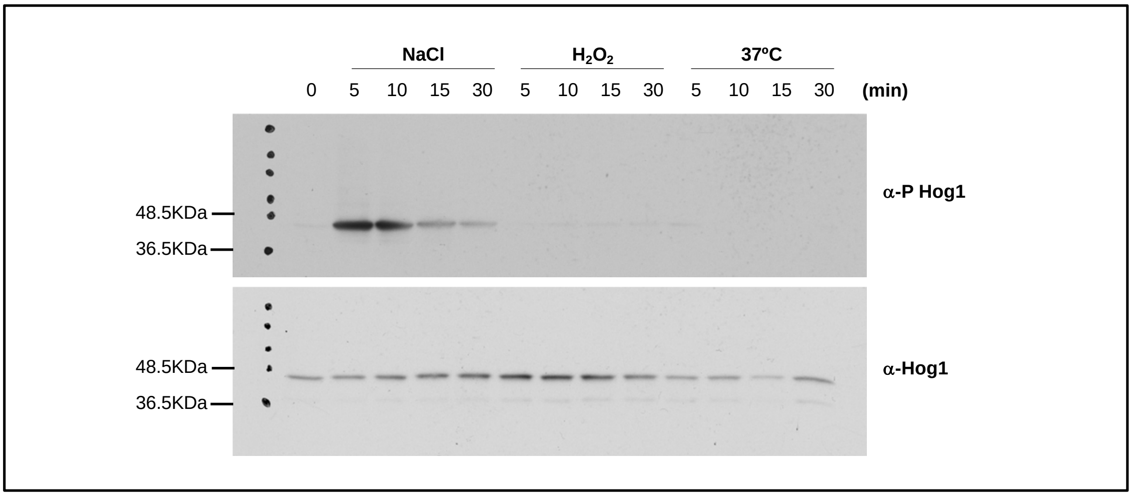

NaCl
H2O2
37ºC
0 5 10 15 30 5 10 15 30 5 10 15 30 (min)
48.5KDa
36.5KDa
α-P Hog1
48.5KDa
36.5KDa
α-Hog1

S4A

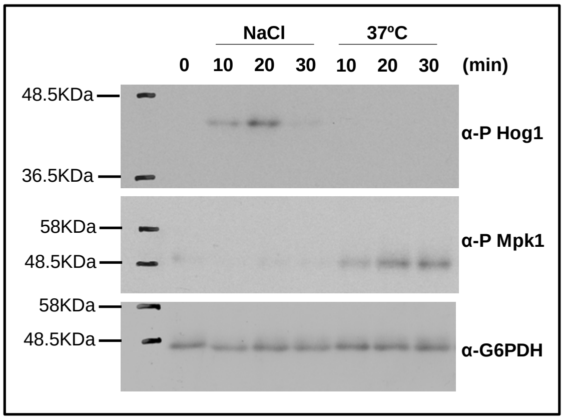

NaCl
37ºC
0 10 20 30 10 20 30 (min)
48.5KDa
36.5KDa
α-P Hog1
58KDa
48.5KDa
α-P Mpk1
58KDa
48.5KDa
α-G6PDH

S5A

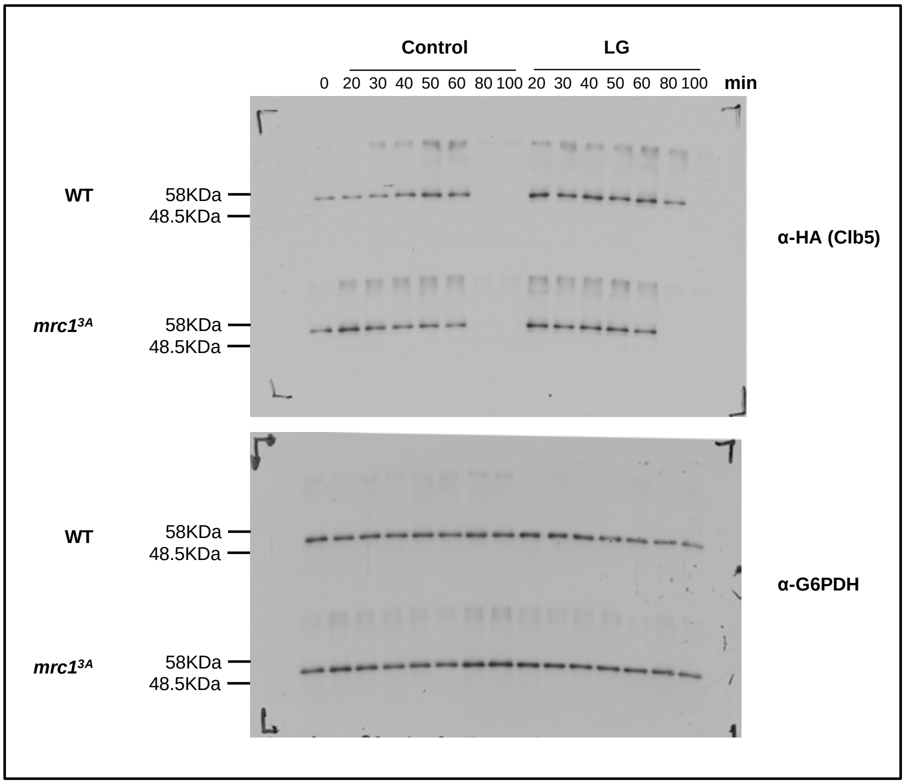

Supplementary Figure 8. Uncropped scans of the blots displayed in the main and supplementary figures.
